# Tracking the role of Aire in immune tolerance to the eye with a TCR transgenic mouse model

**DOI:** 10.1073/pnas.2311487121

**Published:** 2024-01-23

**Authors:** Mianmian Yin, Jennifer A. Smith, Marissa Chou, Jackie Chan, Yingyos Jittayasothorn, Douglas B. Gould, Rachel R. Caspi, Mark S. Anderson, Anthony L. DeFranco

**Affiliations:** ^a^Department of Microbiology and Immunology, University of California, San Francisco, San Francisco, CA 94143; ^b^Diabetes Center, University of California, San Francisco, San Francisco, CA 94143; ^c^Laboratory of Immunology, National Eye Institute, NIH, Bethesda, MD 20892-1857; ^d^Department of Ophthalmology, Institute for Human Genetics, University of California, San Francisco, San Francisco, CA 94143; ^e^Department of Anatomy, Cardiovascular Research Institute, Bakar Aging Research Institute, and Institute for Human Genetics, University of California, San Francisco, San Francisco, CA 94143; ^f^Department of Medicine, University of California, San Francisco, San Francisco, CA 94143

**Keywords:** Aire, autoimmune, negative selection, IRBP, uveitis

## Abstract

Roughly one-half of mice with partial defects in two immune tolerance pathways (Aire^GW/+^Lyn^−/−^ mice) spontaneously develop severe damage to their retinas due to T cell reactivity to Aire-regulated interphotoreceptor retinoid–binding protein (IRBP). Single-cell T cell receptor (TCR) sequencing of CD4^+^ T cells specific for a predominate epitope of IRBP showed a remarkable diversity of autoantigen-specific TCRs with greater clonal expansions in mice with disease. TCR transgenic mice made with an expanded IRBP-specific TCR (P2.U2) of intermediate affinity exhibited strong but incomplete negative selection of thymocytes. This negative selection was absent in IRBP^−/−^ mice and greatly defective in Aire^GW/+^ mice. Most P2.U2^+/−^ mice and all P2.U.2^+/−^Aire^GW/+^ mice rapidly developed inflammation of the retina and adjacent uvea (uveitis). Aire-dependent IRBP expression in the thymus also promoted Treg differentiation, but the niche for this fate determination was small, suggesting differences in antigen presentation leading to negative selection vs. thymic Treg differentiation and a stronger role for negative selection in preventing autoimmune disease in the retina.

## Significance

To model human autoimmune disease, which typically exhibits multigenic inheritance, we created mice with mutations in Aire and Lyn, which respectively alter thymic tolerance of T cells and peripheral activation of T cells. Fifty percent of these mice spontaneously develop eye autoimmunity. TCR transgenic mice were created that recognize the critical retinal autoantigen in order to define more precisely how immune tolerance is compromised. In mice without the Aire mutation, the transgenic T cells almost all die during their thymic development due to recognition of the retinal protein, and a small number become regulatory T cells, which can inhibit disease onset. Upon mutation of Aire, these tolerance mechanisms are lost and this results in strong disease penetrance.

Autoimmune diseases, in which tissue damage results from antibodies or T cells reacting against components of normal tissues, collectively affect over 2% of individuals over their lifespan. In almost all such diseases, a single tissue or type of tissue is attacked, indicating that immune tolerance to self is lost to only one or a few molecular components (“auto-antigens”) (1). Genetic studies indicate that genetic susceptibility to autoimmune disease is strong but, in the vast majority of cases, is due to multiple genetic loci each of which contributes a relatively small degree of risk (2, 3). Correspondingly, autoimmune disease caused by defects in single genes are rare, but they are highly informative about mechanisms of immune tolerance to self. For example, loss of function mutations of Foxp3 in humans or mice causes a rapidly lethal multiorgan autoimmunity due to the absence of regulatory T cells (4–6). Less severe but also highly penetrant is a multiorgan autoimmune syndrome due to either complete loss of function of Aire (7, 8) or to a single copy of a dominant negative mutant allele of Aire, which leaves only small residual Aire function (9). Aire is thought to function primarily by directing expression in the thymus of tissue-specific autoantigens (TSAs), proteins that are ordinarily synthesized in only one tissue (10). Expression of such autoantigens in the thymus can promote tolerance of CD4 T cells by either of two mechanisms: by inducing cells to adopt the regulatory T cell program driven by Foxp3, or by inducing death of the autoreactive T cells in the thymus, a process called negative selection. The relative importance of these two mechanisms of tolerance induction is currently debated (11).

One dominant negative Aire mutant identified in a family in Italy was modeled in mice by making the corresponding mutation (G228W, here referred to as Aire^GW^). This hypomorphic allele of Aire promoted only mild inflammatory disease in salivary and lacrimal glands in C57BL/6 mice, an autoimmune-resistant genetic background, but induced more striking autoimmunity when Aire^GW^ was expressed in non-obese diabetic (NOD) mice (12), a strain of mice with spontaneous autoimmune diabetes resulting from multiple genetic loci (13). These results indicate that a partial loss of thymus-induced tolerance of autoreactive CD4 T cells can cooperate with other genetic susceptibility loci to lead to spontaneous autoimmune disease. Given the complexity of genetic susceptibility in the NOD strain (~20 loci), we sought to model the interaction of multiple autoimmune susceptibility alleles by taking C57BL/6 mice with the Aire^GW/+^ genotype and adding deficiency of Lyn, an intracellular protein tyrosine kinase of the Src family that is important for inhibitory receptor function in dendritic cells, myeloid cells, and B lymphocytes (14, 15). Genetic variation of inhibitory receptors downstream of Lyn may contribute to autoimmune susceptibility for systemic lupus erythematosus (SLE) and other autoimmune diseases (16–19). Lyn deficiency by itself on C57BL/6 results in a lupus-like autoimmunity that starts between 3 and 4 mo of age, but no organ-specific autoimmunity. Remarkably 50% of Aire^GW/+^Lyn^−/−^ mice were found to develop a highly destructive eye autoimmunity in which rod cells of the retina and the adjacent uvea are attacked (referred to as uveitis or uveoretinitis). Fifty percent of mice with these two genetic defects developed uveitis between 6 and 9 wk of age, whereas the remaining mice remained disease-free for as long as they were followed (20). This disease was seen rarely (~3%) in mice with only the Aire dominant negative mutation and not seen in Lyn^−/−^ mice, representing a striking synergy between two autoimmune disease susceptibility mutations. Disease activity correlated strongly with the presence of antibodies and CD4 T cells specific for a rod cell cytoplasmic protein, interphotoreceptor retinoid–binding protein (IRBP), the expression in the thymus of which is under the control of Aire. Moreover, the pathogenic potential of an immune response to IRBP has been shown by immunization of mice with IRBP or with some of its peptide epitopes in adjuvant, which induce experimental autoimmune uveitis (21), and by development of spontaneous uveitis in B10.RIII mice expressing a transgenic TCR specific to a major epitope of IRBP recognized by this strain (22). In addition, in human patients with uveitis, immune responses specific for IRBP have been frequently detected (23), indicating possible relevance to human eye autoimmunity.

In this study, we first tested genetically whether IRBP expression is required for the development of spontaneous uveitis in Aire^GW/+^Lyn^−/−^ mice and found that it was. We then characterized the diversity and sequences of TCRs from CD4 T cells that recognize a dominant epitope from IRBP (amino acids 271 to 290, referred to as the P2 peptide) in the context of the class II MHC I-A^b^. One expanded TCR clonotype of intermediate avidity for the P2/I-A^b^ ligand was chosen to make a TCR transgenic mouse line, called P2.U2. T cells from these transgenic mice recapitulated key elements of this disease model, including the ability to induce destructive uveitis and Aire-dependent central tolerance. For the TCR transgene-expressing thymocytes, Aire-driven expression of IRBP promoted highly efficient deletion of cells at the CD4^+^CD8^−^ stage of development even when they were present at high frequency, and also induced adoption of the Foxp3^+^ T regulatory cell fate when the transgene-expressing thymocytes were present at a low frequency. Thus, autoantigen encounter in the thymus promoted both negative selection and Foxp3 Treg development by the P2.U2 TCR transgenic thymocytes, and the former mechanism of central tolerance was especially robust for thymocytes expressing this TCR of pathogenic potential.

## Results

### IRBP Is Critical for Uveitis in Aire^GW/+^Lyn^−/−^ Mice.

**Fig. 1.**
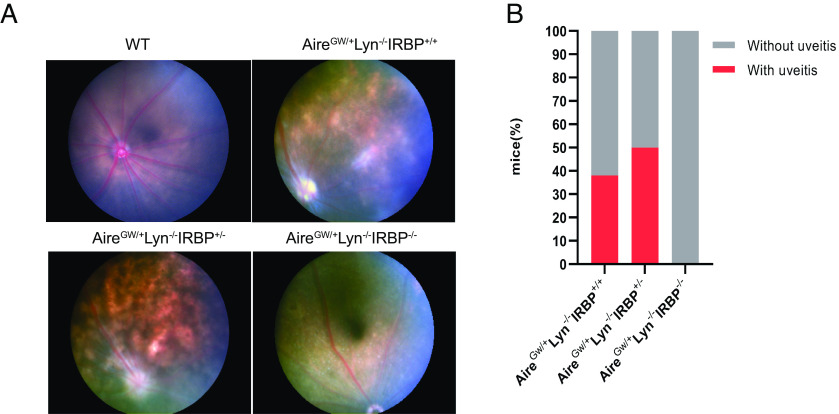
Aire^GW/+^Lyn^−/−^ mice do not develop uveitis in the absence of IRBP. (*A*) Representative funduscopic images of 3-mo-old WT mice without uveitis, Aire^GW/+^Lyn^−/−^IRBP^+/+^ mice with uveitis, Aire^GW/+^Lyn^−/−^IRBP^+/–^ mice with uveitis, and Aire^GW/+^Lyn^−/−^IRBP^−/−^ mice without uveitis. (*B*) Frequencies of Aire^GW/+^Lyn^−/−^IRBP^+/+^ (n = 8), Aire^GW/+^Lyn^−/−^IRBP^+/–^ (n = 10) and Aire^GW/+^Lyn^−/−^IRBP^−/−^ (n = 22) mice with uveitis and without uveitis. Fisher’s exact test, Aire^GW/+^Lyn^−/−^IRBP^+/+^ vs. Aire^GW/+^Lyn^−/−^IRBP^−/−^, *P* = 0.0138; Aire^GW/+^Lyn^−/−^IRBP^+/–^ vs. Aire^GW/+^Lyn^−/−^IRBP^−/−^, *P* = 0.0013.

Previously, we found that approximately 50% of Aire^GW/+^Lyn^−/−^ mice develop uveitis and that disease correlated strongly with a CD4 T cell response to the Aire-regulated retinal protein IRBP (20). To study whether the autoimmune attack in these mice requires the presence of IRBP, we generated Aire^GW/+^Lyn^−/−^IRBP^−/−^ mice and assessed development of uveitis by funduscopy. In agreement with earlier observations, approximately 50% of Aire^GW/+^Lyn^−/−^IRBP^+/–^ or Aire^GW/+^Lyn^−/−^IRBP^+/+^ mice developed uveitis (Fig. 1 *A* and *B*) by 3 mo old age. In contrast, none of the Aire^GW/+^Lyn^−/−^IRBP^−/−^ mice developed uveitis (Fig. 1 *A* and *B*). Therefore, IRBP expression was required for development of uveitis in Aire^GW/+^Lyn^−/−^ mice.

### Expanded TCR Clonotypes in the P2/I-A^b^-Binding CD4^+^T Cells in Aire^GW/+^Lyn^−/−^ Mice.

**Fig. 2.**
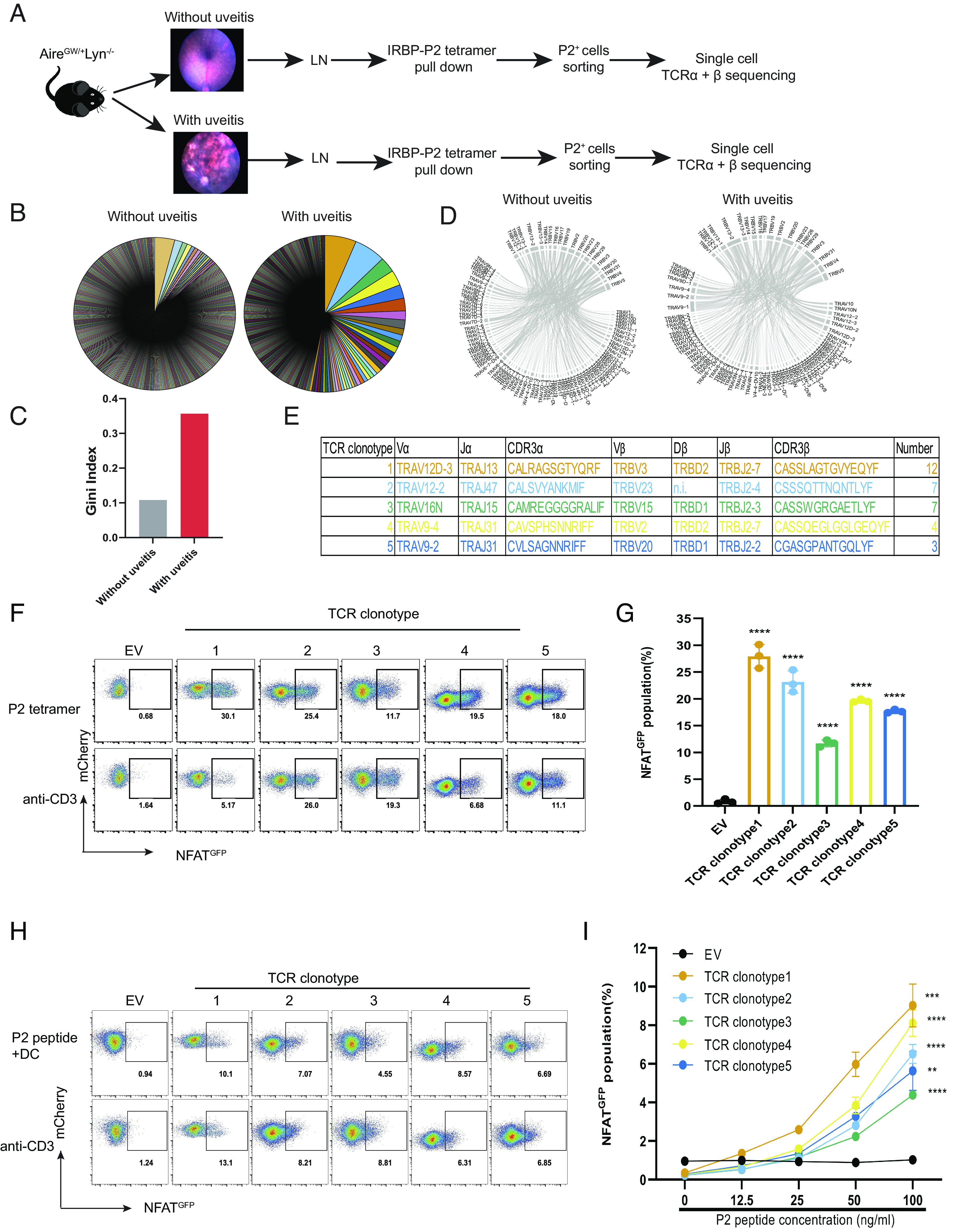
TCR clonotypes of P2/I-A^b^ tetramer-binding CD4^+^ T cells from LN of Aire^GW/+^Lyn^−/−^ mice. (*A*) Experimental schematic. Single-cell suspensions of LN (pooled from eye-draining cervical, submandibular, and axillary lymph nodes) from Aire^GW/+^Lyn^−/−^ mice with or without uveitis were incubated with IRBP-P2 tetramer. P2 tetramer-binding CD4^+^T (P2^+^CD4^+^ T) cells were isolated by magnetic beads enrichment followed by cell sorting and single-cell TCRα and β sequencing. (*B*) Size distributions of TCR clonotypes of P2^+^CD4^+^ T cells in LN of Aire^GW/+^Lyn^−/−^ mice with and without uveitis at 9 wk of age, representing 416 cells in two datasets from seven pooled mice and eight pooled mice, and 380 cells in one dataset from 16 pooled mice, respectively. Cells of the same TCR clonotype are shown as a single pie slice representing the percent of these cells in the entire sample. (*C*) Gini index for TCR clonotypes in the P2^+^CD4^+^T cells in LN of Aire^GW/+^Lyn^−/−^ mice with and without uveitis. (*D*) Chain pairing of TCRVα and TCRVβ from P2^+^CD4^+^ T cells in LN of Aire^GW/+^Lyn^−/−^ mice with and without uveitis are displayed as chord diagrams, where the thickness of ribbons connecting chains indicates the frequency of pairing. (*E*) The V, D, and J segment usage and CDR3 sequences of top five most expanded TCR clonotypes in P2^+^CD4^+^ T cells from Aire^GW/+^Lyn^−/−^ mice with uveitis in dataset 1. n.i.: not identified. (*F*) Representative flow cytometric analysis of NFAT-GFP induction in hybridoma cells expressing empty vector (EV) or TCR clonotypes (1, 2, 3, 4, and 5) after stimulation with P2 tetramer for 24 h. (*G*) Frequency of GFP in (*F*). (*H*) Representative flow cytometric analysis of GFP in hybridomas cells expressing empty vector (EV) or TCRs clonotypes (1, 2, 3, 4, and 5) after stimulation with different doses of P2 peptide in the presence of DC of Lyn^−/−^ mice for 24 h. (*I*) Frequency of GFP in (*H*). **P < 0.01, ***P < 0.001, and ****P < 0.0001. Two-tailed *t* test; error bars are the mean ± SD (n = 3).

Previous results indicated that of three known epitopes of IRBP, the epitope from amino acids 271-290 (referred to as “P2”) was recognized by more CD4 T cells in mice with disease than the other two epitopes (P6 and P7) (20), therefore, we have concentrated our studies on T cells recognizing P2, as detected with a P2/MHC class II tetramer reagent. To characterize the diversity of the spontaneous autoimmune P2 response, CD4 T cells binding to the P2 tetramer were isolated by magnetic bead enrichment followed by cell sorting and subjected to single-cell analysis to determine the sequences of the variable regions of TCRα and TCRβ chains (Fig. 2*A*). Due to the small numbers of P2-specific T cells, multiple mice were pooled in each sample, but they were segregated based on presence or absence of uveitis. The sequencing analysis indicated that there were many different P2-specific clonotypes detected, some of which were expanded in the LN of Aire^GW/+^Lyn^−/−^ mice with uveitis, and to a lesser extent also in LNs from these mice without uveitis (Fig. 2*B*). This difference was reflected in the Gini index, a statistical measure of the degree of expansion in which a higher number indicates more cells with identical TCR variable sequences (Fig. 2*C*). A second method of analyzing variation of TCR usage, iChao, gave a similar result (*SI Appendix*, Fig. S1*A*). There were 338 distinct TCR clonotypes out of 380 TCR in P2-binding (P2^+^CD4^+^) T cells from the LN of Aire^GW/+^Lyn^−/−^ mice without uveitis (Fig. 2*B* and Dataset S1). However, there were 256 distinct TCR clonotypes out of 416 TCR in P2^+^CD4^+^ T cells from the LN of Aire^GW/+^Lyn^−/−^ mice with uveitis (Fig. 2*B* and *SI Appendix*, Fig. S1*A* and Dataset S1), which were obtained in two independent experiments. As shown in Fig. 2*D*, there was a considerable diversity in usage and pairing of the different gene segments encoding TCRα and β variable domains. The two datasets from mice with uveitis contained one identical TCR clonotype, 10 clonotypes with identical CDR3α or CDR3β sequences, and 19 TCR clonotypes with the same paired Vα and Vβ (*SI Appendix*, Fig. S1 *B–E*). The five most expanded clonotypes of dataset 1 (Fig. 2*E*) all shared some elements of TCR V, D, J gene usage or CDR3 sequences with other TCR clonotypes of dataset 1 and dataset 2 (*SI Appendix*, Fig. S1 *F–J*). Thus, although there was a remarkable diversity of TCRs from P2-binding T cells in mice with uveitis, some TCR variable region determinants of expanded TCR clonotypes were seen repeatedly in other clonotypes.

### Expanded TCR Clonotypes Display Strong P2 Antigen Reactivity.

Although the protocol for identifying P2-binding cells has a low background (20), nonetheless, some of the cells analyzed may not be specific for P2, and also some T cells express two different functional TCRα chains, so the TCRα detected in a particular cell may not be responsible for P2 binding. Therefore, we took the top five expanded clonotypes from mice with uveitis and directly tested their specificity for the P2 epitope (Fig. 2*E*). The paired TCRα and β chain sequences were cloned into a retroviral expression vector and introduced into the TCR-deficient 58α-β-hybridoma cell line (Fig. 2*F*). These cells contain an NFAT-GFP reporter so that the reactivity of the transduced TCR for antigen could be detected by induced GFP expression. The TCR clonotype expressing cells were stimulated with plate-bound P2, P6, or P7 tetramers or as a positive control with anti-CD3 antibody for 24 h and GFP expression was assessed. All five tested TCR clonotypes conferred upon the hybridoma cells the ability to respond to the P2 tetramer (Fig. 2 *F* and *G*), but not to the other IRBP epitope tetramers (*SI Appendix*, Fig. S2*A*). The TCR-transduced hybridomas also responded well to LN CD11c^+^ dendritic cells (DCs) incubated with varying doses of P2 peptide (Fig. 2 *H* and *I*) or with intact recombinant IRBP protein (*SI Appendix*, Fig. S2 *B* and *C*). In summary, these results show that these five expanded TCR clonotypes were indeed specific for the P2 epitope and that DCs can present this epitope following uptake of intact IRBP protein.

### TCR Clonotype 2 Transgenic Mice Spontaneously Develop Uveitis.

To facilitate detailed study of the development, tolerization, and pathogenicity of P2-specific CD4 T cells, we made transgenic mice expressing the clonotype 2 TCR driven by T cell-restricted regulatory sequences (*SI Appendix*, Fig. S3*A*). We chose this TCR because 1) TCR clonotype 2 was expanded in conventional P2-specific CD4 T cells in Aire^GW/+^Lyn^−/−^ mice with uveitis, but not found in the Aire^GW/+^Lyn^−/−^ mice without uveitis; 2) TCR clonotype 2 recognized the P2 epitope of IRBP with an intermediate affinity compared to the other tested TCR clonotypes; and 3) TCR clonotype 2 and other TCR clonotypes contained identical TCRβ chain or TCRα CDR3 sequences (*SI Appendix*, Fig. S1*G*).

**Fig. 3.**
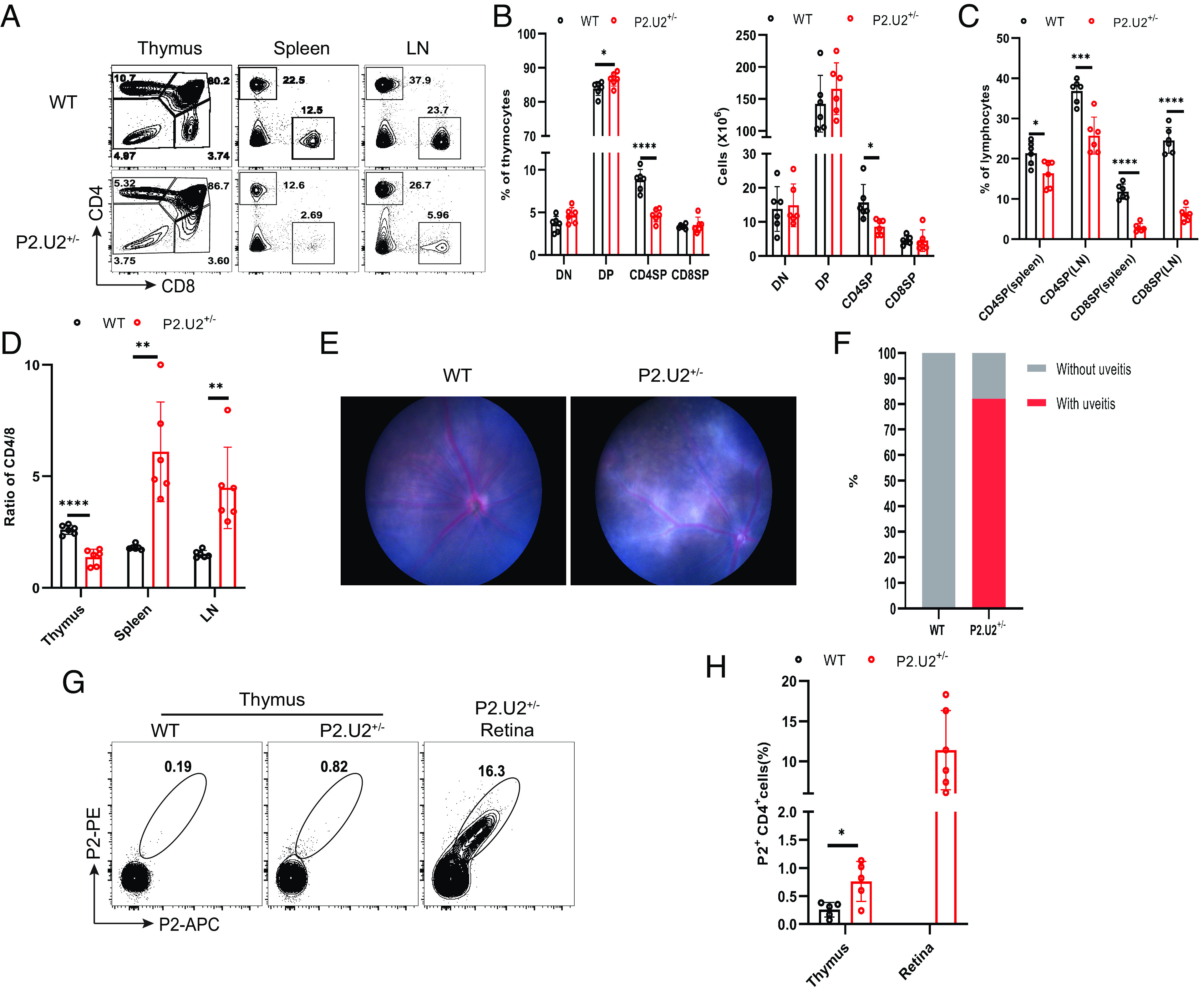
P2.U2^+/−^ transgenic mice spontaneously develop uveitis. (*A*) Representative flow cytometric analysis of CD4 and CD8 in the thymus, spleen and LN of WT (n = 6) or P2.U2^+/−^ (n = 6) mice at 5 to 7 wk, gated on live lymphocytes. (*B*) Frequencies (*Left*) and cell number (*Right*) of CD4^−^CD8^−^(DN), CD4^+^CD8^+^(DP), CD4^+^CD8^−^(CD4SP) and CD4^−^CD8^+^(CD8SP) populations of thymus in panel (*A*). Data were pooled from at least three independent experiments. (*C*) Frequencies of CD4SP and CD8SP populations of spleen and LN in panel (*A*). (*D*) CD4^+^CD8^−^/CD4^−^CD8^+^ ratios in the thymus, spleen, and LN in panel (*B*) and (*C*). (*E*) Representative funduscopic images of 5 to 7 wk old WT mice without uveitis or P2.U2^+/−^ mice with uveitis. (*F*) Frequencies of WT (n = 23) or P2.U2^+/−^ (n = 33) mice with uveitis and without uveitis at 5 to 7 wk. Data are pooled from at least four independent experiments. Fisher’s exact test, P2.U2^+/−^ vs. WT, *P* < 0.0001. (*G*) Representative flow cytometric analysis of P2^+^CD4^+^ T cells in the thymus of WT mice (n = 5) and in the thymus and retina of P2.U2^+/−^ mice (thymus: n = 5; retina n = 6), gated on the TCRβ^+^CD4^+^CD8^−^DUMP^−^ cells. Data were pooled from at least three independent experiments. (*H*) Frequencies of P2^+^CD4^+^ in the thymus or retina in panel (*G*). *P < 0.05; **P < 0.01; ***P < 0.001; ****P < 0.0001. Two-tailed *t* test; error bars are mean ± SD.

In the TCR transgenic mice, hereafter referred to as P2.U2^+/−^ mice, the TCR clonotype 2 TCR α chain (TCRAV12-2) and β chain (TCRBV23) V regions were expressed at an elevated level in the mRNA of thymocytes (*SI Appendix*, Fig. S3*B*). Development of T cells in the thymus of these mice progressed to the single positive stage, but with some reductions in the CD4^+^ and CD8^+^ single positive (SP) populations (Fig. 3 *A* and *B*). In the periphery, there was especially a decrease in the frequency of CD8 T cells (Fig. 3 *C* and *D*). Remarkably, 82% of P2.U2^+/−^ mice spontaneously developed uveitis at 5 to 7 wk of age, despite the absence of mutations in Aire or Lyn (Fig. 3 *E* and *F*). In mice with uveitis, ~12% of the CD4 T cells infiltrating the retina bound to the P2/I-A^b^ tetramer, whereas CD4SP thymocytes bound tetramer with a much lower frequency that was nonetheless elevated compared to non-transgenic thymocytes (Fig. 3 *G* and *H*). As these mice have intact Rag1 and Rag2 genes, we hypothesize that the T cells and CD4SP thymocytes that fail to bind P2 express one or more endogenous TCR chains and hence have a functional TCR other than the transgenic one. Regardless, the P2.U2 transgenic T cells were pathogenic in C57BL/6 mice in the absence of autoimmune susceptibility mutations.

### Lack of Negative Selection of P2-Binding Transgenic T Cells in P2.U2^+/−^IRBP^−/−^ Mice.

**Fig. 4.**
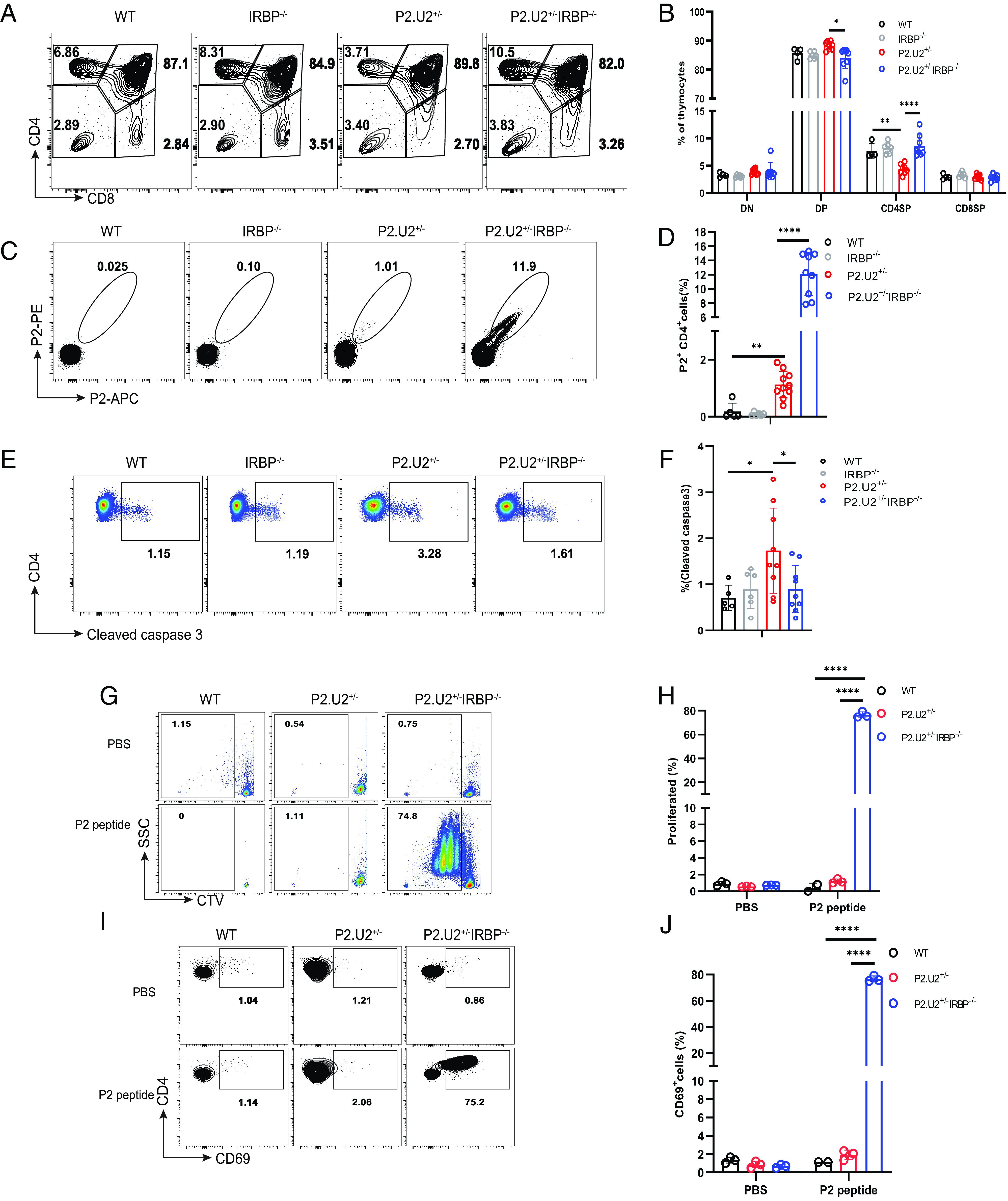
P2.U2 TCR enhances negative selection of CD4SP thymocytes in an IRBP-dependent manner. (*A* and *B*) Representative flow cytometric analysis (*A*) and frequencies (*B*) of T cell subtypes in the thymus of WT (n = 4), IRBP^−/−^ (n = 6), P2.U2^+/−^ (n = 8) and P2.U2^+/−^IRBP^−/−^ (n = 8) mice at 5 to 7 wk of age. (*C* and *D*) Representative flow cytometric analysis (*C*) and frequencies (*D*) of P2^+^ binding by CD4SP thymocytes of WT (n = 5), IRBP^−/−^ (n = 6), P2.U2^+/−^ (n = 10), and P2.U2^+/−^IRBP^−/−^ (n = 9) mice at 5 to 7 wk of age. Cells shown were gated on the TCRβ^+^CD4^+^CD8^−^DUMP^−^ cells. (*E* and *F*) Representative flow cytometric analysis (*E*) and frequencies (*F*) of the presence of cleaved caspase 3 in CD4SP thymocytes of WT (n = 5), IRBP^−/−^ (n = 6), P2.U2^+/−^ (n = 9) and P2.U2^+/−^IRBP^−/−^ (n = 9) mice at 5 to 7 wk. (*G*–*J*) CTV-labeled CD4^+^ T cells from spleens of WT, P2.U2^+/−^, or P2.U2^+/−^IRBP^−/−^mice were cultured in vitro in the presence of PBS or 100 ng/mL P2 peptide with CD11c^+^ dendritic cells (DCs) isolated from the spleen of WT mice. Dilution of CTV and expression of CD69 was assessed by flow cytometry on day 3. (*G* and *H*) Representative flow cytometric analysis (*G*) and frequencies (*H*) of dilution of CTV in CTV-labeled CD4^+^ T cells from WT (PBS: n = 3; P2 peptide: n = 2), P2.U2^+/−^ (n = 3) or P2.U2^+/−^IRBP^−/−^ (n = 3) mice incubated in vitro with PBS or P2 peptide. (*I* and *J*) Representative flow cytometric analysis (*I*) and frequencies (*J*) of expression of CD69 on CTV-labeled CD4^+^ T cells of WT (PBS: n = 3; P2 peptide: n = 2), P2.U2^+/−^ (n = 3) or P2.U2^+/−^IRBP^−/−^ (n = 3) mice stimulated with PBS or P2 peptide. Data were pooled from at least three independent experiments (*A*–*F*) or two independent experiments (*G*–*J*). In (*B*, *F*, *H*, and *J*), one-way ANOVA with Tukey’s multiple comparisons tests were used. In (*D*), two-tailed *t* tests were used. *P < 0.05; **P < 0.01; ****P < 0.0001; error bars are mean ± SD.

One hypothesis to explain the low fraction CD4SP T cells of P2.U2^+/−^ mice that bound to the P2-I-A^b^ tetramer is that most of the thymocytes expressing both transgenic TCRα and TCRβ chains died due to negative selection in the thymus. To test this idea, we bred the TCR transgene to IRBP^−/−^ mice, reasoning that in the absence of IRBP, negative selection would not occur. Indeed, deletion of IRBP led to a twofold increase in the fraction of thymocytes that were CD4SP cells (Fig. 4 *A* and *B*) and a ~12-fold increase in the frequency of these cells that bound to the P2/I-A^b^ tetramer (Fig. 4 *C* and *D*). Direct evidence for IRBP-dependent negative selection in the thymus of these TCR transgenic mice was obtained by intracellular staining of cleaved caspase 3, a marker of apoptosis, in CD4SP thymocytes, which showed an increase in the TCR transgenic mice compared to non-transgenic mice. This increased negative selection was lost upon deletion of IRBP (Fig. 4 *E* and *F*). Consistent with negative selection in the presence of IRBP and not in its absence, P2 tetramer binding of peripheral CD4 T cells was greatly increased by deletion of IRBP, as was the ratio of CD4 T cells to CD8 T cells (*SI Appendix*, Fig. S4). Similarly, the frequency of splenic CD4 T cells that could respond to antigen in vitro, as assessed by CD69 upregulation and proliferation, was greatly increased by deletion of IRBP (Fig. 4 *G*–*J*).

### Decreased Negative Selection of P2.U2 Transgenic T Cells in Aire^GW/+^ Mice.

**Fig. 5.**
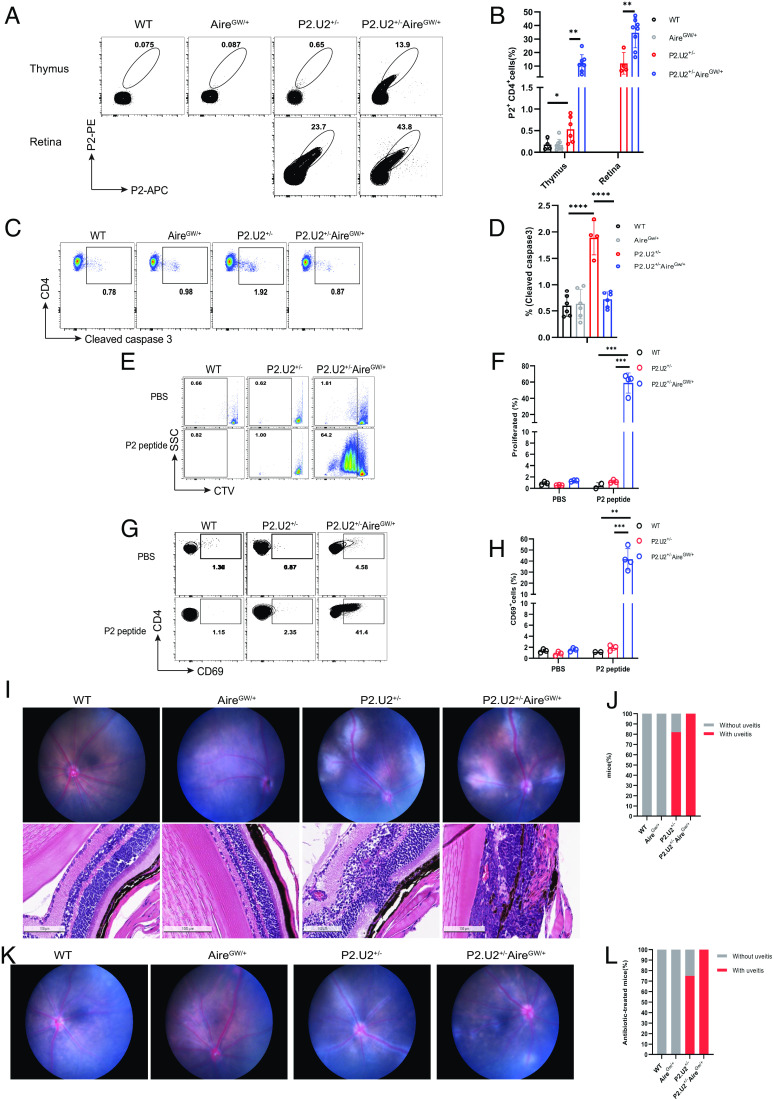
Aire^GW/+^ mice have greatly reduced negative selection of P2.U2 TCR transgenic T cells. (*A* and *B*) Representative flow cytometric analysis (*A*) and frequencies (*B*) of P2^+^CD4^+^ T cells in the thymus, gated on TCRβ^+^CD4^+^CD8^−^DUMP^−^thymocytes, and retina, gated on TCRβ^+^CD4^+^CD8^−^DUMP^−^T cells, of WT (n = 4), Aire^GW/+^ (n = 8), P2.U2^+/−^ (thymus: n = 6; retina: n = 4) and P2.U2^+/−^Aire^GW/+^ (n = 8) mice at 5 to 7 wk of age. Data were pooled from at least three independent experiments. For thymus data of the P2.U2^+/−^ group of panel (*B*), two of the mice were also included in the data shown in (Fig. 3*H*). (*C* and *D*) Representative flow cytometric analysis (*C*) and frequencies (*D*) of cleaved caspase 3 staining in CD4^+^CD8^-^ thymocytes of WT (n = 4), Aire^GW/+^ (n = 6), P2.U2^+/−^ (n = 4) and P2.U2^+/−^Aire^GW/+^ (n = 6) mice at 5 to 7 wk. Data were pooled from at least three independent experiments. (*E*–*H*) CTV-labeled splenic CD4^+^ T cells from WT, P2.U2^+/−^, or P2.U2^+/−^Aire^GW/+^ mice were cultured in vitro with CD11c^+^ DCs, isolated from the spleens of WT mice, in the presence of PBS or 100 ng/mL P2 peptide. Dilution of CTV and expression of CD69 was assessed by flow cytometry on day 3. Representative flow cytometric analysis (*E* and *G*) and frequencies (*F* and *H*) of dilution of CTV (*E* and *F*) and CD69 induction (*G* and *H*) in CTV-labeled splenic CD4^+^ T cells from WT (n = 3), P2.U2^+/−^ (n = 3) or P2.U2^+/−^Aire^GW/+^ (n = 4) mice. Data were pooled from two independent experiments. For the data of the WT and P2.U2^+/−^ groups of panels (*F* and *H*), the data is the same for these groups as shown in Fig. 4 *H* and *J*. (*I*) Representative funduscopic images (*Top* row) or H&E-stained retinal sections (*Bottom* row) for 2- to 4-mo-old WT, Aire^GW/+^ mice without uveitis, and P2.U2^+/−^, P2.U2^+/−^Aire^GW/+^ mice with uveitis. (*J*) Frequencies of uveitis or lack of uveitis in WT (n = 23), Aire^GW/+^ (n = 16), P2.U2^+/−^ (n = 33) and P2.U2^+/−^Aire^GW/+^ (n = 37) mice at 5 to 7 wk. Fisher’s exact test, P2.U2^+/−^ vs. WT, *P* < 0.0001; P2.U2^+/−^Aire^GW/+^vs. WT, *P* < 0.0001; P2.U2^+/−^ vs. P2.U2^+/−^Aire^GW/+^, *P* = 0.0084. For WT and P2.U2^+/−^ group of Fig. 5*J*, the data is the same as the WT and P2.U2^+/−^ groups of Fig. 3*F*. (*K* and *L*) Representative funduscopic images (*K*) and frequencies (*L*) of WT (n = 3), Aire^GW/+^ (n = 16), P2.U2^+/−^ (n = 12) and P2.U2^+/−^Aire^GW/+^ (n = 4) mice with uveitis or without uveitis after antibiotic treatment for 6 to 9 wk starting at birth. Fisher’s exact test, P2.U2+/− vs. WT, *P* = 0.044; P2.U2^+/−^Aire^GW/+^ vs. WT, *P* = 0.0286. In (*B*), two-tailed *t* tests were used. In (*D*, *F*, and *H*), one-way ANOVA with Tukey’s multiple comparisons tests were used. *P < 0.05; **P < 0.01; ***P < 0.001; ****P < 0.0001; error bars are mean ± SD.

Whereas IRBP^−/−^ mice completely lack IRBP expression, Aire^GW/+^ mice express IRBP normally in the retina, but have approximately 10% of normal IRBP expression in the thymus (12). Therefore, we next examined the effect of the Aire^GW/+^ genotype on the negative selection of P2.U2 transgenic thymocytes. Remarkably, P2.U2 CD4SP thymocytes had reduced negative selection in the thymus, similarly to IRBP-deficient mice (Fig. 5). This was evident in the frequency of cleaved caspase 3 positive CD4SP thymocytes, as well as the frequency of these cells that bound to the P2-tetramer reagent (Fig. 5 *A*–*D*). In P2.U2^+/−^IRBP^−/−^ or P2.U2^+/−^Aire^GW/+^ mice, the CD4SP thymocytes had equivalent CD5 expression levels and this was toward the high end of CD5 expression in diverse TCR repertoire CD4SP thymocytes (*SI Appendix*, Fig. S6 *A–E*), presumably reflecting higher than average avidity for positively selecting self-peptides other than those derived from IRBP. In addition, an elevated fraction of peripheral CD4 T cells in these mice bound to the P2-tetramer (*SI Appendix*, Fig. S5 *A–I*) and also responded to P2 in vitro as indicated by induced expression of CD69 and proliferation (Fig. 5 *E*–*H*). As shown above, the peripheral P2-specific CD4 T cells from transgenic mice with normal Aire and IRBP were poorly responsive to antigen in vitro, but some of these cells had induced high levels of CD44 in vivo, suggesting that they had responded to IRBP as elevated CD44 expression was not seen in transgenic P2-specific T cells from IRBP^−/−^ mice (*SI Appendix*, Fig. S6 *F* and *G*). All P2.U2^+/−^Aire^GW/+^ mice rapidly developed uveitis by 5 to 7 wk of age (Fig. 5 *I* and *J*), in contrast to the P2.U2^+/−^IRBP^−/−^ mice, which did not. To detect whether gut commensal microbes might play a role in the spontaneous uveitis of P2.U2^+/−^ and P2.U2^+/−^Aire^GW/+^ mice, P2.U2^+/−^ and P2.U2^+/−^Aire^GW/+^ mice were treated with a broad-spectrum antibiotic cocktail (MGVCK: metronidazole, gentamicin, vancomycin, colistin, and kanamycin) in drinking water from birth until 9 wk of age. Around 75% P2.U2^+/−^ and all P2.U2^+/−^Aire^GW/+^ mice developed uveitis at 6 to 9 wk age with MGVCK treatment (Fig. 5 *K* and *L*). Disease severity did not seem to be altered (*SI Appendix*, Fig. S5*J*). This indicates that signals from commensal bacteria were not required for uveitis development in P2.U2^+/−^ and P2.U2^+/−^Aire^GW/+^ mice, in contrast to what has been reported with the R161H TCR transgenic mouse, which is specific for a different epitope of IRBP (24). This is similar to what has been reported for autoimmunity in Aire^−/−^ mice (25) and Aire^GW/+^ Lyn^−/−^ mice (20).

### A Small Minority of P2.U2 Transgenic T Cells Can Adopt a Treg State If IRBP Is Expressed Normally in the Thymus.

**Fig. 6.**
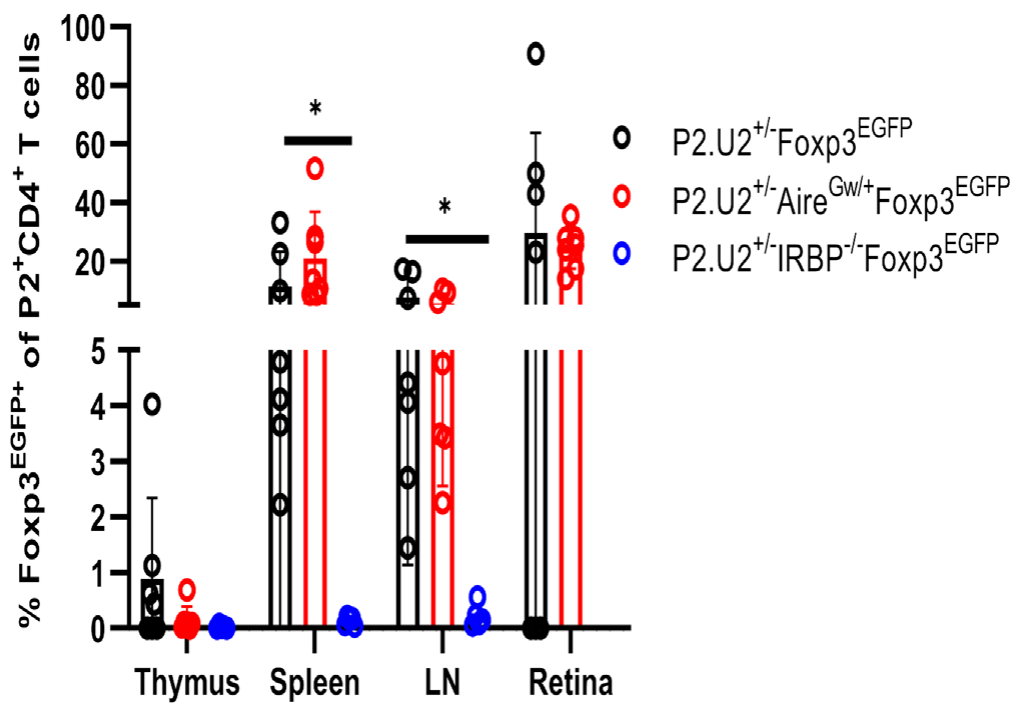
A minority of P2.U2 TCR transgenic T cells specific for P2 tetramer adopt a Treg fate in the thymus of mice that express IRBP, but are enriched in the periphery. Frequencies of Foxp3^EGFP+^ of P2^+^ CD4^+^ T cells in the thymus, spleen, LN and retina of P2.U2^+/−^Foxp3^EGFP^ (n = 7), P2.U2^+/−^Aire^GW/+^Foxp3^EGFP^ (n = 7) and P2.U2^+/−^IRBP^−/−^Foxp3^EGFP^ (n = 5) mice at 6 to 7 wk of age. Cells shown were gated on TCRβ^+^CD4^+^CD8^−^DUMP^−^P2^+^ cells. Data were pooled from at least three independent experiments. Two-tailed *t* test was used, *P < 0.05; error bars are mean ± SD.

It is thought that Aire can promote tolerance to TSAs by at least two mechanisms, promotion of negative selection of CD4SP thymocytes and/or induction of Treg fate determination (10, 11). To facilitate analysis of possible Treg differentiation of P2.U2 transgenic thymocytes, we introduced a Foxp3-EGFP reporter allele and bred P2.U2^+/−^IRBP^−/−^Foxp3^EGFP^ and P2.U2^+/−^Aire^GW/+^Foxp3^EGFP^ mice. In the presence of normal IRBP expression in the thymus, a small fraction, less than 1% of P2 tetramer-binding transgenic CD4SP thymocytes expressed Foxp3-EGFP. An even smaller fraction, possibly representing experimental background, of P2 tetramer-binding transgenic T cells expressed Foxp3-EGFP in P2.U2^+/−^ mice deficient for IRBP or expressing a hypomorphic Aire genotype (Fig. 6 and *SI Appendix*, Fig. S7*A*). Foxp3-EGFP induction in CD4SP thymocytes that did not bind to the P2 tetramer was higher and was not affected by mutations in IRBP or Aire (*SI Appendix*, Fig. S7 *B* and *C*). Compared with CD4SP thymocytes, a much higher fraction of spleen and lymph node P2 tetramer-binding CD4 T cells expressed Foxp3-EGFP in mice expressing IRBP and with normal or hypomorphic Aire function. This result suggests that recognition of the self-antigen in eye-draining LN and/or in the retina can enhance the numbers of Treg specific for IRBP P2, either by peripheral induction of Treg fate or by promoting the survival and/or proliferation of the small number of thymically derived Treg of this specificity. In addition, in the TCR transgenic mice with uveitis, a substantial fraction of the P2-tetramer binding cells in the retina expressed Foxp3-EGFP (Fig. 6 and *SI Appendix*, Fig. S7*A*).

### The Niche for Treg Commitment of P2.U2^+^ Thymocytes Is Limited.

**Fig. 7.**
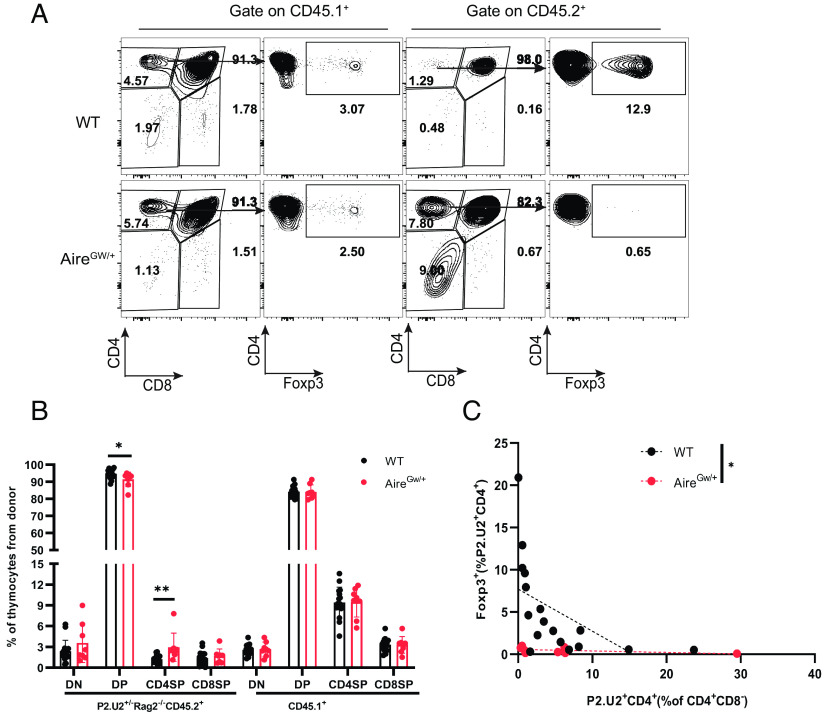
A small niche for thymic development of IRBP P2-specific Tregs is present in Aire-expressing mice. Mixed bone marrow chimeric mice were made in which bone marrow cells of P2.U2^+/−^Rag2^−/−^CD45.2^+^ male donor mice were engrafted along with various fractions of non-transgenic bone marrow cells from male CD45.1^+^ mice. Recipient mice were irradiated WT or Aire^GW/+^CD45.1^+^/CD45.2^+^ mice. Six weeks post-engraftment, the fate of P2.U2^+^ T cells was analyzed. (*A*) Representative flow cytometric analysis of CD45.1^+^polyclonal T cells (*Left*) and P2.U2^+^Rag2^−/−^CD45.2^+^ T cells (*Right*) from recipient mice of the indicated Aire genotype receiving a small percentage of CD45.2^+^ bone marrow (WT recipient:1.93% CD45.2^+^ cells; Aire^GW/+^:0.43% CD45.2^+^ cells). The left column (CD4 vs. CD8) depicts all cells in the lymphocyte gate; the right column (intracellular Foxp3 vs. CD4) depicts CD4^+^CD8^−^ gated samples. (*B*) Frequencies of DN, DP, CD4SP and CD8SP populations of thymocytes for donor cells (P2.U2^+^Rag2^−/−^CD45.2^+^ cells and CD45.1^+^ cells) in WT (n = 16) or Aire^GW/+^CD45.1^+^/CD45.2^+^ (n = 8) recipient mice in panel (*A*). (*C*) Summary plot of the efficiency of P2.U2^+^ Treg development, in which the frequencies of P2.U2^+/−^Rag2^−/−^CD45.2^+^ cells that express Foxp3 is plotted vs. the frequency of P2.U2^+/−^Rag2^−/−^CD45.2^+^CD4^+^ thymocytes in all donor CD4^+^CD8^−^ cells isolated from recipient mice of the indicated genotype. Dashed lines indicate simple linear regression line [WT (n = 17): R = 0.298; Aire^GW/+^ (n = 9): R = 0.249]. *P < 0.05; **P < 0.01. In (*B* and *C*), two-tailed *t* test; error bars are mean ± SD.

Whereas P2.U2 transgenic T cells exhibited striking negative selection in the thymus in an Aire-dependent fashion, adoption of a Treg fate in the thymus was limited to a small fraction of the P2 tetramer-binding cells. However, previous studies with a TCR transgenic system in which the autoantigen was an Aire-regulated gene from the prostate (Tcaf3), indicated that the niche for Treg fate determination in the thymus medulla may be easily saturated in the context of a TCR transgenic mouse (26). Therefore, to examine Treg fate determination of P2.U2 thymocytes in the context of a more physiological precursor frequency, we used a mixed bone marrow chimeric mouse approach. Chimeric animals were made in which bone marrow cells from either P2.U2^+/−^Rag2^−/−^CD45.2^+^ (Fig. 7 *A–C* and *SI Appendix*, Fig. S8*B*) or P2.U2^+/−^CD45.2^+^ (*SI Appendix*, Fig. S8 *K–M*) donors were mixed with different proportions of wild-type CD45.1^+^ bone marrow cells and engrafted into lethally irradiated WT or Aire^GW/+^ CD45.1^+^/CD45.2^+^ recipient mice. By using P2.U2^+/−^Rag2^−/−^CD45.2^+^ transgenic donor bone marrow, CD45.2 could be used to track P2-specific transgenic CD4SP thymocytes, as such cells must express both transgenic TCR chains. A fraction of the P2.U2^+/−^Rag2^−/−^ transgenic mice spontaneously developed uveitis (*SI Appendix*, Fig. S8*A*), as was seen with the P2.U2^+/−^ transgenic mice. After engraftment, the fractions of CD4SP T cells expressing Foxp3 were assessed in the two donor types of thymocytes and of peripheral T cells (*SI Appendix*, Fig. S8 *E–G*). The efficiency of Treg development of transgenic P2.U2 cells in the thymus of the mixed bone marrow chimeric mice was dependent upon Aire function and was inversely correlated with the frequency of P2.U2^+^ precursors in the thymus (Fig. 7 *A*–*C* and *SI Appendix*, Fig. S8 *K*–*M*). As the P2.U2^+^ CD4SP thymocytes were subjected to negative selection, there was some concern that using this population as the relevant precursor to Treg might be problematic, particularly if the niche for negative selection were limited. To address this point, we considered P2.U2^+^CD45.2^+^ DP thymocytes as an alternative measure for the number of P2.U2^+^ thymocytes that had the potential for becoming Treg in the thymus. Therefore, we graphed these data as total number of P2.U2^+/−^ Rag2^−/−^CD45.2^+^CD4^+^ CD8^-^ Foxp3^+^ Treg thymocytes vs. number of potential precursors (P2.U2^+/−^Rag2^−/−^CD45.2^+^DP thymocytes) and although there was scatter, it appeared that there was a linear increase in P2.U2^+^ Treg with precursor number until that number reached roughly 10 million cells, after which the increase in Treg number with increased numbers of precursors appeared to be small (*SI Appendix*, Fig. S8*C*). We also graphed the number of P2.U2 ^+/−^ Rag2^−/−^CD45.2^+^CD4^+^ CD8^−^ thymocytes against the number of P2.U2^+/−^Rag2^−/−^CD45.2^+^DP thymocytes in the mixed bone marrow chimeric mice (*SI Appendix*, Fig. S8*D*) and a close linear relationship was seen if the recipients were Aire^GW/^*^+^*, that is, in mice without negative selection for this TCR. In the presence of negative selection (WT recipients), there was again a linear relationship until 25 million DP precursors, although possibly deviation from the line after that. The difference between the two lines is perhaps due to negative selection, in which case negative selection may have saturated at roughly 25 million CD45.2^+^ DP thymocytes, or about 2.5× higher DP thymocytes compared to saturation for Treg differentiation. The primarily linear relationship between these two transgenic populations also indicated that using either population was suitable to assess saturation of the Treg developmental niche for the P2.U2^+^ TCR transgenic cells. The fraction of CD4SP CD45.1^+^ control cells that became Treg was not affected by either Aire genotype or frequency of P2U2^+^ thymocytes (*SI Appendix*, Fig. S8 *M* and *N*), as expected as they have a diverse TCR repertoire and many Treg differentiate in the thymus of Aire-deficient mice (27). Three out of nine chimeric mice made with Aire^GW/+^CD45.1^+^/CD45.2^+^ recipients developed spontaneous uveitis (*SI Appendix*, Fig. S8*J*), but were not characterized by a deficiency of polyclonal Tregs (*SI Appendix*, Fig. S8*N*). None of 17 WT CD45.1^+^/CD45.2^+^ recipient mice developed spontaneous uveitis (Fisher’s exact test, WT vs. Aire^GW/+^: *P* = 0.0323), indicating that immune tolerance to IRBP was efficiently achieved in the wild-type recipients, whereas it was incompletely achieved in Aire^GW/+^ recipients.

Examination of development of the P2.U2^+/−^Rag2^−/−^CD45.2^+^ thymocytes in the mixed bone marrow chimeric mice showed that the transgenic TCRβ chain was able to efficiently drive maturation to the CD4CD8 DP stage of thymocyte development and that the transgenic TCRα chain was able to promote maturation to the CD4SP stage of development (Fig. 7 *A* and *B*). The proportion of CD45.2^+^ thymocytes that were CD4SP was substantially lower than the proportion for the control CD45.1^+^ thymocytes, suggesting that positive selection promoted by the transgenic TCR-expressing CD4CD8DP thymocytes was somewhat less efficient for some reason, even in Aire^GW/+^ recipients where negative selection was minimal. Despite this caveat, in the absence of IRBP expression in the thymus, transgenic TCR expression in Rag2^−/−^ or Rag^+^ thymocytes was reasonably good as judged by the intensities of P2-I-A^b^ tetramer staining (*SI Appendix*, Fig. S8 *H* and *I* and Fig. 4*C*, respectively). Analysis of the CD45.2^+^ transgenic thymocytes in the two types of recipients also supported the previous conclusion that Aire-induced expression of IRBP promoted negative selection of CD4SP thymocytes, as there was an increased frequency of CD45.2^+^CD4SP thymocytes positive for intracellular staining of cleaved caspase 3 in WT compared to Aire^GW/+^ recipient mice (*SI Appendix*, Fig. S8 *O* and *P*).

## Discussion

In order to gain insight into the mechanism of spontaneous autoimmune uveitis development in Aire^GW/+^Lyn^−/−^ mice, we characterized the TCRs of CD4 T cells specific for a key autoantigen epitope in the eye-draining lymph nodes of mice with and without uveitis and made a transgenic mouse line that re-capitulates key aspects of this disease model using the TCR from one expanded clonotype of intermediate avidity from mice with disease. Previous studies had implicated in disease pathogenesis a CD4 T cell response to IRBP, resulting from a defect in T cell tolerance to this Aire-regulated, cytoplasmic rod cell protein combined with enhanced activation of autoreactive T cells in the periphery due to loss of Lyn function in dendritic cells (20). Here, we confirmed the key role of IRBP in disease initiation by showing that genetic deletion of it prevented uveitis development in Aire^GW/+^Lyn^−/−^ mice, as is also the case for Aire^−/−^ mice (28). We then focused on T cells recognizing the P2 epitope of IRBP, as these cells were previously found to be more abundant in mice with uveitis than CD4 T cells recognizing the other two known epitopes of IRBP in C57BL/6 mice (20). Paired TCRα and TCRβ V(D)J sequences were obtained from P2 tetramer-binding CD4 T cells from the eye-draining lymph nodes of mice with or without uveitis. Surprisingly, these TCRs exhibited a great diversity, regardless of disease status, although there was a greater number of expanded clones in mice with uveitis. TCRs from the five most abundant clonotypes from mice with disease were tested, and all were able to confer functional responsiveness to IRBP in transfected T hybridoma cell lines. An expanded clonotype TCR with intermediate avidity for the P2 epitope was chosen to make TCR transgenic mice, referred to as P2.U2^+/−^ mice. The transgenic T cells in mice with normal IRBP expression in the thymus exhibited a high degree of negative selection, although a small number of autoantigen-specific T cells escaped to the periphery and rapidly caused destructive uveitis in the majority of these mice. These results indicated that P2.U2 TCR transgenic T cells had a substantial pathogenic potential. In contrast, TCR transgenic mice with hypomorphic Aire function (Aire^GW/+^) had much reduced negative selection and all of these mice developed uveitis. Thus, Aire has a strong role in tolerizing P2.U2 transgenic T cells, with negative selection in the thymus being especially prominent as a mechanism.

While Aire-dependent negative selection of the P2-specific P2.U2 TCR transgenic thymocytes was clearly evident in the transgenic mice with a high frequency of thymocytes specific for P2, there was also a small fraction of TCR transgenic thymocytes that adopted a Treg fate, and this was also dependent upon Aire function. These results are in agreement with current thinking that Aire-driven expression of tissue-restricted autoantigens in medullary thymic epithelial cells is capable of driving both negative selection and Treg differentiation (11). Remarkably, the fraction of P2-specific transgenic T cells that exhibited a Treg fate was much higher in peripheral T cells than in CD4SP thymocytes and in the periphery was equivalent in Aire^+^ and Aire^GW/+^ mice. It is not clear at this time whether these peripheral Treg represented expansion and/or survival of the small number of P2-specific Treg that developed in the thymus or whether these Treg represented post-thymic Treg differentiation in the context on an autoimmune response. As Aire^GW/+^ P2.U2 transgenic mice and Aire^+^ P2.U2 transgenic mice had these cells in equal proportions, peripheral Treg differentiation is perhaps a more likely explanation. In any case, the small fraction of P2-specific TCR transgenic thymocytes that adopted a Treg differentiation state was a reflection of an Aire-dependent thymic niche for this fate that was much smaller than the niche for negative selection. The limited size of the thymic niche for Treg differentiation of P2.U2 transgenic thymocytes was revealed by making mixed bone marrow chimeric mice with a small fraction of the bone marrow coming from the transgenic mice, thereby reducing the precursor frequency of P2-specific thymocytes. In this context, 20% or more of the live P2-specific TCR transgenic T cells adopted a Treg fate provided the recipient was Aire^+^. In contrast, robust negative selection was clearly evident in mice with a high proportion of thymocytes expressing the transgenic TCR. These results suggest that for the P2.U2 TCR transgenic T cells, Aire-dependent tolerance induction involves both negative selection and Treg differentiation. In the context of a high precursor frequency, e.g., in the TCR transgenic mice, negative selection was incompletely efficient and Treg fate determination was limited by a small niche size, resulting in escape to the periphery of enough P2-specific conventional CD4 T cells to initiate autoimmune disease in the majority of mice. When the frequency of the P2.U2 transgenic T cells was reduced in the mixed bone chimeric mouse experiments, autoimmune attack on the retina still occurred in 3/9 Aire^GW/+^ recipients and was not seen in any out of 17 Aire^+^ recipients, again emphasizing a key role for Aire in preventing autoimmune uveitis in the context of an additional challenge to immune tolerance, in this case, an increased frequency of autoreactive CD4 T cells.

P2.U2 TCR transgenic mice have several features that are distinct from previously described TCR transgenic mice recognizing Aire-regulated tissue-restricted autoantigens. Horai et al. (22) made IRBP-specific TCR transgenic mice against a different IRBP peptide in B10.RIII mice, a strain that is highly susceptible to experimental autoimmune uveitis following immunization with IRBP. Three transgenic lines with different levels of expression with the same TCR were made, and the lines with the higher levels of expression (R161H and R161M) spontaneously developed uveitis in B10RIII mice. A small fraction of the TCR-expressing cells in the thymus expressed Foxp3, consistent with what was seen with P2.U2 TCR transgenic thymocytes. Whether or not R161H or R161M TCR-expressing thymocytes exhibited negative selection in the thymus is not clear, but thymic generation of Treg was limited, similarly to our current findings (22). Remarkably, R161H mice developed severe uveitis only when a normal gut microbiota was present, and moreover, in mice with a normal gut microbiota, the R161H transgenic T cells in the gut lamina propria had an activated effector phenotype (24). Thus, the R161H transgenic mice appear to be a situation where the self-reactive T cells require activation by a microbe-derived cross-reactive epitope, a situation referred to as molecular mimicry. In contrast, P2.U2 mice developed uveitis at similar frequencies in untreated mice or in mice treated to greatly reduce gut microbiota, using an antibiotic cocktail treatment similar to the one used by Horai et al. (24). We previously found that presentation of the P2/I-A^b^ epitope of IRBP by DCs in the eye-draining LNs is readily detectable (20), and moreover, in Aire^GW/+^Lyn^−/−^ mice without disease, we detected an expansion of P2-specific CD4 T cells specifically in these LN, so it is possible that P2.U2 transgene-expressing CD4 T cells can be activated in that location and then traffic to the eye to induce inflammation. Additional experiments will be required to address this point further.

In addition, central tolerance of P2.U2 TCR transgenic T cells was clearly distinct from that observed with two TCR transgenic strains recognizing different epitopes of an Aire-regulated prostate autoantigen called Tcaf3. MJ23 and SP33 TCRs were originally identified in Treg from Aire^+^ mice and used to make TCR transgenic mice (26, 29). Thymocytes expressing these TCRs did not show evidence of negative selection but were efficiently induced to become Treg in the thymus of Aire^+^ mice, and therefore, tolerance for these two specificities appears to be primarily dependent on Treg commitment in the thymus (30, 31). As with P2.U2 TCR-expressing thymocytes, MJ23 and SP33 TCR-expressing thymocytes had a small niche for Treg development, which only was efficient at inducing Treg commitment in the context of reduced numbers of TCR transgenic thymocytes, as in mixed bone marrow chimeric mice (31). Thus, Aire mediates immune tolerance of Tcaf3-specific MJ23 and SP33 transgenic T cells and of IRBP-specific P2.U2 transgenic T cells by largely distinct mechanisms: primarily by Treg fate direction in the case of MJ23 and SP33, and by negative selection for P2.U2, with possibly some contribution of Treg differentiation.

We envision two possible non-exclusive explanations for the striking difference in the mechanism of Aire-driven central tolerance between the two Tcaf3-specific TCR transgenic strains and the IRBP-specific P2.U2 TCR transgenic strain. The first relates to differences in the experimental approach used to identify the different TCRs. Whereas P2.U2 was identified in conventional CD4 T cells from mice with a genetic defect in Aire function, MJ23 and SP33 were identified in Treg from Aire^+^ mice, an approach that may bias against TCRs that efficiently undergo negative selection in wild-type mice. A second possible explanation derives from the observation that P2.U2 TCR-expressing thymocytes exhibited strong negative selection even in the transgenic mice with a high precursor frequency, whereas the niche for Treg development in the thymus was small, as was also the case for Treg differentiation of MJ23 and SP33. These results suggest that the P2/I-A^b^ epitope may be presented by two different cell types in the thymus, one that induces negative selection and a second that is capable of inducing Treg fate commitment. According to this hypothesis, Tcaf3 epitopes recognized by MJ23 and SP33 may only be presented by the latter type of antigen-presenting cell, which has been shown to be DCs (32). Such a difference between IRBP and Tcaf3 (or certain epitopes from them) could reflect distinct cell biological or biochemical properties of these two tissue-restricted proteins. Consistent with the latter hypothesis is that analysis of diverse T cells binding to a tetramer made with the epitope recognized by MJ23 indicated that Aire-dependent tolerance to this epitope in non-transgenic mice was primarily due to Treg determination, not negative selection (31).

In summary, we describe here the creation of a TCR transgenic mouse strain, P2.U2 mice, representing an expanded clonotype in the draining LN of Aire^GW/+^Lyn^−/−^ mice with spontaneous uveitis and specific for a predominate epitope of the key retinal autoantigen IRBP. T cells expressing this transgene recapitulated key aspects of spontaneous autoimmune eye disease in Aire^GW/+^Lyn^−/−^ mice, namely Aire-dependent negative selection of P2-specific CD4 T cells (12, 20) and pathogenic potential of P2-specific conventional CD4 T cells. T cells from three prior TCR transgenic strains of mice recognizing IRBP or the Aire-regulated prostate-specific protein, Tcaf3, all shared some properties with P2.U2 T cells, including potential for Treg differentiation in the thymus and autoimmune pathogenic potential. However, P2.U2 T cells also exhibited striking differences from the other three transgenic T cells, namely strong negative selection in the thymus compared to the lack of negative selection of Tcaf3-specific thymocytes, and the lack of cross-reactivity with a component of the microbiota, which was seen with the R161H transgenic T cells and which was largely required for their pathogenicity. These differences raise interesting questions for future studies for which P2.U2 will be useful, including whether antigen-presenting cells in the thymus directing negative selection vs. Treg differentiation are distinct, and what is the mechanism leading to activation of the pathogenic potential of autoreactive conventional CD4 T cells that are not cross-reactive with the microbiota. Thus, the P2.U2 TCR transgenic mouse model has provided insights into mechanisms of immune tolerance to self and is likely to provide additional insights upon further investigation.

## Materials and Methods

### Mice.

Aire^GW/+^ Lyn^−/−^ mice used in this study were previously described (20). B6.129P2-Rbp3 tm1Gil/J(IRBP^−/−^)(stock no. 023080), B6(Cg)-Rag2^tm1.1Cgn^/J(Rag2^−/−^)(stock no 008449), C.Cg-Foxp3^tm2Tch^/J(Foxp3^EGFP^)(stock no 006769), and B6.SJL-Ptprc^a^ Pepc^b^/BoyJ (stock no 002014) mice were obtained from the Jackson Laboratory. All animal experiments were approved by the UCSF Animal Care and Use Committee. P2.U2^+/−^ mice were generated by the transgenic core facility of the Gladstone Institutes, San Francisco, by microinjecting pCD2 TCR clonotype 2α chain and p428 TCR clonotype 2β chain DNA into C57BL/6 (Envigo, Strain, C57BL/6NHsd) embryos. Aire^GW/+^ or WT CD45.1^+^/ CD45.2^+^ were from breeding of Aire^GW/+^ with B6.SJL-Ptprc^a^ Pepc^b^/BoyJ mice.

Intestinal microbes were depleted as previously described (33). The drinking water with MGVCK was given to pregnant dams and continued after weaning until mice were 9 wk of age.

Ocular funduscopy was performed as previous described (20) by using a Micron III camera (Phoenix Research Labs Inc.).

### Flow Cytometry.

Single-cell suspensions of the thymus, spleen (with red blood cell lysis), LN, or disrupted retina were washed, blocked with purified Anti-CD16/32 Antibody (BioLegend, 101302, clone 93), and stained with antibodies of indicated specificities in HBSS buffer with 2% FBS, and, where indicated, with Allophycocyanin (APC) or phycoerythrin(PE)-conjugated I-A^b^ P2 tetramer (QTWEGSGVLPCVG) corresponding to mouse IRBP amino acids 277 to 290, obtained from the NIH Tetramer Facility. All data were collected on an LSR II cytometer (BD) or LSRFortessa (BD) at the Flow Cytometry Core at UCSF and analyzed by using FlowJo software (TreeStar). For additional details, see *SI Appendix*, *Materials and Methods*.

### Single-Cell TCR Sequencing and Reconstitution into Hybridoma Cells.

For single-cell TCR variable region sequencing, P2 tetramer-specific T were isolated as described previously (20) and loaded onto the 10× Genomics Chromium platform for droplet-based massively parallel scRNA-seq according to the manufacturer’s instructions. The Seurat pipeline was used to cluster and identify the cell subsets with the dataset. Clonotypes were defined as multiple cells with identical TRA and TRB sequences at the nucleotide level.

TCRα and β variable domain-encoding regions from TCR clonotypes 1,2,3,4,5 of dataset 1 were cloned into the pMSCV-IRES-mCherry retroviral vector (Addgene) as previously described (34, 35), and used to transfect the TCR-deficient 58α-β-hybridoma cell line, which has an NFAT promoter-driven GFP reporter. TCRβ^+^mCherry^+^ cells were enriched by cell sorting and stimulated as described.

### Generation of P2.U2^+/−^ Transgenic Mice.

The TCR variable region sequences corresponding to clonotype 2 TCRα chain were cloned into the pCD2 vector, which contains the human CD2 promoter (36). The TCRβ chain variable region sequence was cloned into the p428 vector, which contains the CD4 promoter (37). The linearized plasmids were microinjected together into C57BL/6 embryos to generate multiple transgenic founders, one of which was selected for further analysis and referred to as P2.U2. More details are included in *SI Appendix*, *Materials and Methods*. For stimulation of TCR transgenic T cells, CD4^+^ T cells were obtained by magnetic bead isolation, labeled with CellTrace™ Violet (CTV) (ThermoFisher, C34571), and cultured with splenic dendritic cells in vitro.

### Bone Marrow (BM) Chimeric Mice.

Bone marrow cells from tibia and femur of 6 to 8 wk P2.U2^+/−^Rag2^−/−^CD45.2^+^ or P2.U2^+/−^CD45.2^+^ donor mice were engrafted, along with various fractions of non-transgenic “helper” bone marrow cells from CD45.1^+^mice, into irradiated WT or Aire^GW/+^CD45.1^+^/CD45.2^+^recipient mice. All recipient mice were irradiated twice with 550 rad. Recipients were humanely killed by CO_2_ narcosis from a gas source after 6 wk.

### Statistics.

Statistical analysis and graphing were performed by using Prism 9.3.1 (GraphPad). Statistical tests for comparison of two groups involved two-tailed *t* tests, and comparisons of multiple groups used ordinary one-way ANOVA with Tukey’s multiple comparisons tests. The number of independent experiments, statistical tests, and *P* values are indicated in figure legends.

## Supplementary Material

Appendix 01 (PDF)

Dataset S01 (XLSX)

## Data Availability

Data for single sell sequencing and single-cell TCR sequencing have been deposited at Gene Expression Omnibus (GEO) database (https://www.ncbi.nlm.nih.gov/geo) (38) and are publicly available as of the date of publication. The accession number is GSE216402.

## References

[1] C. C. Goodnow, Multistep pathogenesis of autoimmune disease. Cell **130**, 25–35 (2007). 17632054 10.1016/j.cell.2007.06.033

[2] J. H. Cho, P. K. Gregersen, Genomics and the multifactorial nature of human autoimmune disease. N Engl. J. Med. **365**, 1612–1623 (2011). 22029983 10.1056/NEJMra1100030

[3] S. Pillai, Rethinking mechanisms of autoimmune pathogenesis. J. Autoimmun. **45**, 97–103 (2013). 23809879 10.1016/j.jaut.2013.05.003 PMC4820393

[4] S. Hori, T. Nomura, S. Sakaguchi, Control of regulatory T cell development by the transcription factor Foxp3. Science **299**, 1057–1061 (2003). 12522256 10.1126/science.1079490

[5] R. Khattri, T. Cox, S. A. Yasayko, F. Ramsdell, An essential role for Scurfin in CD4^+^CD25^+^ T regulatory cells. Nat. Immunol. **4**, 337–342 (2003). 12612581 10.1038/ni909

[6] J. D. Fontenot, M. A. Gavin, A. Y. Rudensky, Foxp3 programs the development and function of CD4^+^CD25^+^ regulatory T cells. Nat. Immunol. **4**, 330–336 (2003). 12612578 10.1038/ni904

[7] K. Nagamine , Positional cloning of the APECED gene. Nat. Genet. **17**, 393–398 (1997). 9398839 10.1038/ng1297-393

[8] A. C. Finnish-German, An autoimmune disease, APECED, caused by mutations in a novel gene featuring two PHD-type zinc-finger domains. Nat. Genet. **17**, 399–403 (1997). 9398840 10.1038/ng1297-399

[9] B. E. Oftedal , Dominant mutations in the autoimmune regulator AIRE are associated with common organ-specific autoimmune diseases. Immunity **42**, 1185–1196 (2015). 26084028 10.1016/j.immuni.2015.04.021

[10] M. S. Anderson, M. A. Su, AIRE expands: New roles in immune tolerance and beyond. Nat. Rev. Immunol. **16**, 247–258 (2016). 26972725 10.1038/nri.2016.9 PMC4831132

[11] L. Klein, E. A. Robey, C. S. Hsieh, Central CD4(+) T cell tolerance: Deletion versus regulatory T cell differentiation. Nat. Rev. Immunol. **19**, 7–18 (2019). 30420705 10.1038/s41577-018-0083-6

[12] M. A. Su , Mechanisms of an autoimmunity syndrome in mice caused by a dominant mutation in Aire. J. Clin. Invest. **118**, 1712–1726 (2008). 18414681 10.1172/JCI34523 PMC2293336

[13] D. V. Serreze , Initiation of autoimmune diabetes in NOD/Lt mice is MHC class I-dependent. J. Immunol. **158**, 3978–3986 (1997). 9103469

[14] Y. Xu, K. W. Harder, N. D. Huntington, M. L. Hibbs, D. M. Tarlinton, Lyn tyrosine kinase: Accentuating the positive and the negative. Immunity **22**, 9–18 (2005). 15664155 10.1016/j.immuni.2004.12.004

[15] P. Scapini, S. Pereira, H. Zhang, C. A. Lowell, Multiple roles of Lyn kinase in myeloid cell signaling and function. Immunol. Rev. **228**, 23–40 (2009). 19290919 10.1111/j.1600-065X.2008.00758.x PMC3248569

[16] V. W. Chan, F. Meng, P. Soriano, A. L. DeFranco, C. A. Lowell, Characterization of the B lymphocyte populations in Lyn-deficient mice and the role of Lyn in signal initiation and down-regulation. Immunity **7**, 69–81 (1997). 9252121 10.1016/s1074-7613(00)80511-7

[17] C. Lamagna, P. Scapini, J. A. van Ziffle, A. L. DeFranco, C. A. Lowell, Hyperactivated MyD88 signaling in dendritic cells, through specific deletion of Lyn kinase, causes severe autoimmunity and inflammation. Proc. Natl. Acad. Sci. U.S.A. **110**, E3311–3320 (2013). 23940344 10.1073/pnas.1300617110 PMC3761623

[18] C. Lamagna, Y. Hu, A. L. DeFranco, C. A. Lowell, B cell-specific loss of Lyn kinase leads to autoimmunity. J. Immunol. **192**, 919–928 (2014). 24376269 10.4049/jimmunol.1301979 PMC3900234

[19] K. G. Smith, M. R. Clatworthy, FcgammaRIIB in autoimmunity and infection: Evolutionary and therapeutic implications. Nat. Rev. Immunol. **10**, 328–343 (2010). 20414206 10.1038/nri2762 PMC4148599

[20] I. Proekt , LYN- and AIRE-mediated tolerance checkpoint defects synergize to trigger organ-specific autoimmunity. J. Clin. Invest. **126**, 3758–3771 (2016). 27571405 10.1172/JCI84440 PMC5087700

[21] R. R. Caspi, A look at autoimmunity and inflammation in the eye. J. Clin. Invest. **120**, 3073–3083 (2010). 20811163 10.1172/JCI42440 PMC2929721

[22] R. Horai , Breakdown of immune privilege and spontaneous autoimmunity in mice expressing a transgenic T cell receptor specific for a retinal autoantigen. J. Autoimmun **44**, 21–33 (2013). 23810578 10.1016/j.jaut.2013.06.003 PMC3743101

[23] F. J. Descamps , Interphotoreceptor retinoid-binding protein as biomarker in systemic autoimmunity with eye inflictions. J. Cell Mol. Med. **12**, 2449–2456 (2008). 18266969 10.1111/j.1582-4934.2008.00264.x PMC4514122

[24] R. Horai , Microbiota-dependent activation of an autoreactive T cell receptor provokes autoimmunity in an immunologically privileged site. Immunity **43**, 343–353 (2015). 26287682 10.1016/j.immuni.2015.07.014 PMC4544742

[25] D. H. Gray, I. Gavanescu, C. Benoist, D. Mathis, Danger-free autoimmune disease in Aire-deficient mice. Proc. Natl. Acad. Sci. U.S.A. **104**, 18193–18198 (2007). 17991771 10.1073/pnas.0709160104 PMC2084319

[26] S. Malchow , Aire-dependent thymic development of tumor-associated regulatory T cells. Science **339**, 1219–1224 (2013). 23471412 10.1126/science.1233913 PMC3622085

[27] S. Yang, N. Fujikado, D. Kolodin, C. Benoist, D. Mathis, Immune tolerance. Regulatory T cells generated early in life play a distinct role in maintaining self-tolerance. Science **348**, 589–594 (2015). 25791085 10.1126/science.aaa7017 PMC4710357

[28] J. DeVoss , Spontaneous autoimmunity prevented by thymic expression of a single self-antigen. J. Exp. Med. **203**, 2727–2735 (2006). 17116738 10.1084/jem.20061864 PMC2118158

[29] J. D. Leonard , Identification of natural regulatory T cell epitopes reveals convergence on a dominant autoantigen. Immunity **47**, 107–117.e108 (2017). 28709804 10.1016/j.immuni.2017.06.015 PMC5562039

[30] S. Malchow , Aire enforces immune tolerance by directing autoreactive T cells into the regulatory T cell lineage. Immunity **44**, 1102–1113 (2016). 27130899 10.1016/j.immuni.2016.02.009 PMC4871732

[31] D. E. J. Klawon , Altered selection on a single self-ligand promotes susceptibility to organ-specific T cell infiltration. J. Exp. Med. **218**, e20200701 (2021). 33914024 10.1084/jem.20200701 PMC8091134

[32] D. S. Leventhal , Dendritic cells coordinate the development and homeostasis of organ-specific regulatory T cells. Immunity **44**, 847–859 (2016). 27037189 10.1016/j.immuni.2016.01.025 PMC4842258

[33] A. T. Stefka , Commensal bacteria protect against food allergen sensitization. Proc. Natl. Acad. Sci. U.S.A. **111**, 13145–13150 (2014). 25157157 10.1073/pnas.1412008111 PMC4246970

[34] S. H. Krovi, J. W. Kappler, P. Marrack, L. Gapin, Inherent reactivity of unselected TCR repertoires to peptide-MHC molecules. Proc. Natl. Acad. Sci. U.S.A. **116**, 22252–22261 (2019). 31570608 10.1073/pnas.1909504116 PMC6825295

[35] A. Spence , Revealing the specificity of regulatory T cells in murine autoimmune diabetes. Proc. Natl. Acad. Sci. U.S.A. **115**, 5265–5270 (2018). 29712852 10.1073/pnas.1715590115 PMC5960284

[36] T. Zhumabekov, P. Corbella, M. Tolaini, D. Kioussis, Improved version of a human CD2 minigene based vector for T cell-specific expression in transgenic mice. J. Immunol. Methods **185**, 133–140 (1995). 7665895 10.1016/0022-1759(95)00124-s

[37] S. Sawada, J. D. Scarborough, N. Killeen, D. R. Littman, A lineage-specific transcriptional silencer regulates CD4 gene expression during T lymphocyte development. Cell **77**, 917–929 (1994). 8004678 10.1016/0092-8674(94)90140-6

[38] M. Yin , Tracking the role of Aire in immune tolerance to the eye with a TCR transgenic mouse model. GEO database. https://www.ncbi.nlm.nih.gov/geo. Deposited 24 October 2022. 10.1073/pnas.2311487121 PMC10835137 38261611

