# Supplementary material for: Tracking the role of Aire in immune tolerance to the eye with a TCR transgenic mouse model
Source: Proc Natl Acad Sci U S A. 2024 Jan 23;121(5):e2311487121. doi: 10.1073/pnas.2311487121 (PMC10835137; doi:10.1073/pnas.2311487121)
Supplement: Supplementary file 1 — Appendix 01 (PDF) [file pnas.2311487121.sapp.pdf]

Supplementary materials and methods

Antibiotic treatment of mice

Intestinal microbes were depleted as previously described (1) by giving mice drinking water with a broad-spectrum antibiotic cocktail (MGVCK), consisting of metronidazole (0.043 mg/ml, Sigma-Aldrich, PHR1052), gentamicin (0.007 mg/ml, Sigma-Aldrich, G1914), vancomycin (0.5 mg/ml, Sigma-Aldrich, V2002), colistin (170 U/ml, Sigma-Aldrich, C4461) and kanamycin (0.08 mg/ml, VWR International, LLC, 97061) to deplete both aerobic and anaerobic commensal bacteria. The drinking water with MGVCK was given to pregnant dams and continued after weaning until mice were 9 weeks of age.

Funduscopy

Ocular funduscopy was performed as previous described (2) by using a Micron III camera (Phoenix Research Labs Inc.). Mice were anesthetized with isoflurane. Eye drops containing Phenylephrine hydrochloride 2.5%(Sigma Pharmaceuticals, LLC, 3020-1) and Tropicamide ophthalmic 1%(Sigma Pharmaceuticals, LLC, 3035-1) were used to relax eye muscles and dilate the pupil. To avoid dryness of the cornea, TheraTears (Sigma Pharmaceuticals, LLC, 5035) and GenTeal® Gel (Sigma Pharmaceuticals, LLC, 5009-1) were used during imaging. Presence of uveitis and disease severity was determined according to previously described grading system (3).

Histology

Eyes of mice were harvested and fixed overnight in 10% formalin, washed in 30% ethanol for 30 minutes, and then stored in 70% ethanol. Fixed eyes were embedded in paraffin, sectioned, and stained with H&E by HistoWiz, Inc. Scoring of eye immune infiltration were done as previously described in a blinded fashion (4). Presence of uveitis was determined according to the histological findings.

Lymphocytes from retina

Briefly, both retinas were dissected and transferred to digestion buffer (RPMI 1640 medium with 10% FBS). Retinas were minced with scissors and digested in digestion buffer containing 0.5 mg/ml collagenase D (Roche Diagnostics Deutschland GmbH, 11088858001) and 100 U/ml DNase I (Roche Diagnostics Deutschland GmbH, 10104159001) for 1 hour at 37°C. Retinal lymphocytes were purified by centrifugation through a 30%/37%/70% Percoll (Cytiva, 17-5445-02) step gradient for further analysis.

Flow cytometry

Single-cell suspensions of thymus, lymphocytes of retina, spleen (with red blood cell lysis) and LN were washed, blocked with purified Anti-CD16/32 Antibody (BioLegend, 101302, clone 93), and stained with antibodies of indicated specificities in HBSS buffer with 2% FBS. Staining reagents include APC-eFluor 780 Anti-TCR beta (47-5961-82, clone H57-597) from Thermo Fisher Scientific, purified Anti-CD3 (70-0032, clone 17A2) and PE-Cyanine7 Anti-CD4 (60-0042, clone RM4-5) from Tonbo biosciences, purified Anti-CD16/32 Antibody (101302, clone 93), Pacific Blue™ Anti-CD19 (115523, clone 6D5), Pacific Blue™ Anti-F4/80 (123124, clone BM8), Pacific Blue™ Anti-CD11b (101224, clone M1/70), Pacific Blue™ Anti-CD11c (117322, clone N418), APC/Cyanine7 Anti-CD69(104526, clone H1.2F3), PerCP/Cy5.5 Anti-CD45.1 (110727, clone A20), FITC Anti-CD45.2 (109806, clone 104) from Biolegend, PE Anti-FOXP3 (12-5773-80, clone FJK16s) from eBioscience™, BV605 Anti-CD8 (563152, clone 53-6.7) and Alexa Fluor® 647 Rabbit Anti-Active Caspase-3 (560626, clone C92-605) from BD bioscience. Dead cell exclusion was based on DAPI(Thermo fisher Scientific, D1306) or 7-AAD staining (BD bioscience, 559925). For intracellular staining, cells were stained using Foxp3/Transcription Factor staining Buffer set (Thermo Fisher Scientific, 00-5123-43, 00-8333-56, 00-5223-56) according to the manufacturer's protocol. Dead cell exclusion was based on LIVE/DEAD staining (Thermo Fisher Scientific, L34962).

Allophycocyanin(APC) or phycoerythrin(PE)-conjugated I-A^b P2 tetramer (QTWEGSGVLPCVG) corresponding to mouse IRBP amino acids 277–290, phycoerythrin-conjugated I-A^b P7 tetramer (SYSSAVPLLCSY) corresponding to mouse IRBP amino acids 771–782, and phycoerythrin-conjugated I-A^b P6 tetramer (GAYRTAVDLES) corresponding to mouse IRBP amino acids 654–664 were obtained from the NIH tetramer facility, Atlanta, Georgia, USA.

For flow cytometry to characterize P2-specific T cells, single cells from thymus, retina, spleen (with red blood cell lysis) and LN were incubated with tetramer in staining buffer (2%FBS, 10ug/ml purified Anti-CD16/32 antibody, 5% normal mouse serum(Jackson ImmunoResearch Inc, 015-000-120) and 5% normal rat serum) for 1 hour at room temperature. The cells were stained, and tetramer-reactive cells were gated on $TCR\beta^+CD4^+CD8^-$ $DUMP^-$($CD19^-CD11b^-CD11c^-F4/80^-$) lymphocytes for further analysis. All data were collected on an LSR II cytometer (BD) or LSRFortessa (BD) at the Flow Cytometry Core at UCSF and analyzed by using FlowJo software (TreeStar).

Cell culture

Phoenix-ECO cells and 58α-β-hybridoma cell line were cultured in complete DMEM (Sigma D6546) Supplemented with 10% FBS, Pen/Strept (Gibco,15140122), 50 uM 2-mercaptoethanol (Gibco,21985023), and Glutamax (Gibco, 35050061).

Single cell TCR sequencing

For single cell TCR variable region sequencing, P2 tetramer-specific T were isolated as described previously (2). Briefly, single-cell suspensions of LN were incubated with tetramer for 1 hour at room temperature in staining buffer (2% FBS, 10ug/ml

purified anti-CD16/32 antibody, 5% normal mouse serum and 5% normal rat serum) and PE and APC-conjugated I-A^b P2 tetramer, followed by magnetic bead enrichment for tetramer-positive cells with Anti-PE (Miltenyi Biotec, 130-048-801) and Anti-APC magnetic beads (Miltenyi Biotec, 130-090-855). The positively selected cells were stained, and tetramer-reactive cells were gated on $TCR\beta^+CD4^+CD8^-DUMP^-(CD19^-CD11b^-CD11c^-F4/80^-)$ lymphocytes for sorting and single cell TCR sequence determination. Enriched and sorted $P2^+$ cells were loaded onto the 10x Genomics Chromium platform for droplet-based massively parallel scRNA-seq according to the manufacturer's instructions by the genomics core of UCSF. According to the manufacturer's protocol (Illumina), libraries were prepared using the 10x Genomics Chromium Single Cell 5′ Reagent (GEX + VDJ) Kit version 1.1 (10xGenomics, PN-1000165, PN-1000020, PN-1000005, PN-1000120, PN-1000213) and sequenced using an Illumina Novaseq 6000. FASTQ were aligned using the Cell Ranger pipeline (10x Genomics Inc., version 3) using mouse genome reference dataset (mm10) or VDJ reference dataset (GRCm38_alts_ensembl). Clonotypes were defined as multiple cells with identical TRA and TRB sequences at the nucleotide level. Cells with unique TRA and TRB combinations were referred to as clonotypes with 1 representative. For the results shown in Fig. 2, only the cells with a full complement of TRαV, TRαJ, TRβV, TRβJ, CDR3_α, and CDR3_β were included. TCR clonotypes were counted within each sample, and expanded TCR clonotypes were defined where more than one cell was assigned to the TCR clonotype.

TCR-transfected hybridoma cell generation and stimulation

TCRα and β variable domain-encoding regions from TCR clonotypes 1,2,3,4,5 of dataset 1 were cloned into the pMSCV-IRES-mCherry retroviral vector (Addgene) as previously described (5, 6). TCR clonotypes expressing retroviruses were produced using Phoenix-ECO packaging cells, concentrated using Retro-X™ Concentrator (Takara, 631455), and used to transfect the TCR-deficient 58α-β-hybridoma cell line, which has an NFAT promoter-driven GFP reporter. Seventy-two hours after infection, $TCR\beta^+mCherry^+$ cells were enriched by cell sorting. $2X10^4$ enriched hybridoma cells per well (96 well plates) were stimulated with either plate bound anti-CD3 antibody (well coated with clone 17A2, Tonbo Biosciences) or with plate bound P2, P7 and P6 tetramer of IRBP (coated with 2.5ug/mL). Cells were stimulated overnight in complete DMEM at 37°C and GFP expression was analyzed by flow cytometry after 24 hours. Alternatively, DCs of LN from $Lyn^{-/-}$ mice were enriched by binding to anti-CD11c magnetic beads (Miltenyi, 130-125-835), eluted, and plated in 96-well tissue culture plates ($8X10^4$/well). TCR-expressing hybridoma cells and various concentrations of P2 peptide (Genemed Synthesis) or intact recombinant IRBP protein were added to the DCs and GFP induction was analyzed by flow cytometry.

Cloning of P2-specific TCR clonotypes and generation of $P2.U2^{+/-}$ mice

The TCR clonotype 2α chain was cloned into the pCD2 vector, which contains the human CD2 promoter(7). The TCR clonotype 2β chain was cloned into the p428 vector, which contains the CD4 promoter(8). pCD2 TCR clonotype 2α chain DNA was

linearized with SalI and NotI. p428 TCR clonotype 2β chain DNA was linearized with NotI. Linearized pCD2 TCR clonotype 2α chain and p428 TCR clonotype 2β chain DNAs were microinjected together into C57BL/6 embryos. Founder mice carrying both TCR clonotype 2α chain and TCR clonotype 2β chain were tested for expression of the transgenic TCR, and a founder with good expression was chosen for analysis and breeding to mice with relevant genetic mutations. The genotyping primers for TCR clonotype 2α chain were: Forward primer: TGCTTTGAGTGTCTATGCAAAC; Reverse primer: AAATCCGGCTACTTTCAGCAG. The genotyping primers for TCR clonotype 2β chain were: Forward primer: TATGTAGCACTTTGCCTCCTGG; Reverse primer: TCTCAGATCCTCTAGCACCGAT.

Quantitative PCR

RNA was extracted using TRIzol (Thermo Fisher Scientific) reagent according to manufacturer's protocol. cDNA was synthesized by reverse transcriptase with SuperScript III enzyme (Fisher Scientific). Quantitative PCR was performed in the ABI Step One System (Applied Biosystems) with SYBR™ Green PCR Master Mix (Thermo Fisher Scientific, 4344463). mRNA expression levels of genes were normalized to GAPDH mRNA expression. Quantitative PCR primers for AV12-2: Forward: TGACACCTGCTCAGTTCTTGT; Reverse: GTTCTGGATGTGAGGTCTGACT. Quantitative PCR primers for BV23: Forward: TATGTAGCACTTTGCCTCCTGG; Reverse: TCTCAGATCCTCTAGCACCGAT.

In Vitro stimulation of TCR transgenic T cells

$CD4^+$T cells were isolated from spleen of $P2.U2^{+/-}$, $P2.U2^{+/-}Aire^{GW/+}$, $P2.U2^{+/-}IRBP^{-/-}$ or WT mice and purified by using EasySep™ Mouse $CD4^+$ T Cell Isolation Kit(STEMCELLTechnologies Inc., 19852), according to the manufacturer instructions. $CD4^+$T cells were labeled with CellTrace™ Violet (CTV) (ThermoFisher, C34571) by centrifuging purified $CD4^+$T cells and resuspending them in PBS with CTV at a 1:2,000 dilution, followed by incubation for 20 min at room temperature in the dark. Remaining unreacted dye was inactivated by the addition of 10 mL of complete tissue culture medium. To isolate dendritic cells (DCs) from spleen of C57BL/6 WT mice, splenocytes were incubated with $CD11c^+$ magnetic beads (Miltenyi Biotech, 130-125-835) and then separated magnetically. $2x10^4$ CTV-labeled $CD4^+$T cells were co-cultured with $8x10^4$ $CD11c^+$ DCs, 100ng/ml P2 peptide or PBS in tissue culture medium as indicated. Dilution of CTV and expression of CD69 was detected by flow cytometry analysis on day 3.

References

1. A. T. Stefka *et al.*, Commensal bacteria protect against food allergen sensitization. *Proc Natl Acad Sci U S A* **111**, 13145-13150 (2014).

2. I. Proekt *et al.*, LYN- and AIRE-mediated tolerance checkpoint defects synergize to trigger organ-specific autoimmunity. *J Clin Invest* **126**, 3758-3771 (2016).
3. H. Xu *et al.*, A clinical grading system for retinal inflammation in the chronic model of experimental autoimmune uveoretinitis using digital fundus images. *Exp Eye Res* **87**, 319-326 (2008).
4. M. A. Su *et al.*, Mechanisms of an autoimmunity syndrome in mice caused by a dominant mutation in Aire. *J Clin Invest* **118**, 1712-1726 (2008).
5. S. H. Krovi, J. W. Kappler, P. Marrack, L. Gapin, Inherent reactivity of unselected TCR repertoires to peptide-MHC molecules. *Proc Natl Acad Sci U S A* **116**, 22252-22261 (2019).
6. A. Spence *et al.*, Revealing the specificity of regulatory T cells in murine autoimmune diabetes. *Proc Natl Acad Sci U S A* **115**, 5265-5270 (2018).
7. T. Zhumabekov, P. Corbella, M. Tolaini, D. Kioussis, Improved version of a human CD2 minigene based vector for T cell-specific expression in transgenic mice. *J Immunol Methods* **185**, 133-140 (1995).
8. S. Sawada, J. D. Scarborough, N. Killeen, D. R. Littman, A lineage-specific transcriptional silencer regulates CD4 gene expression during T lymphocyte development. *Cell* **77**, 917-929 (1994).

Fig. S1

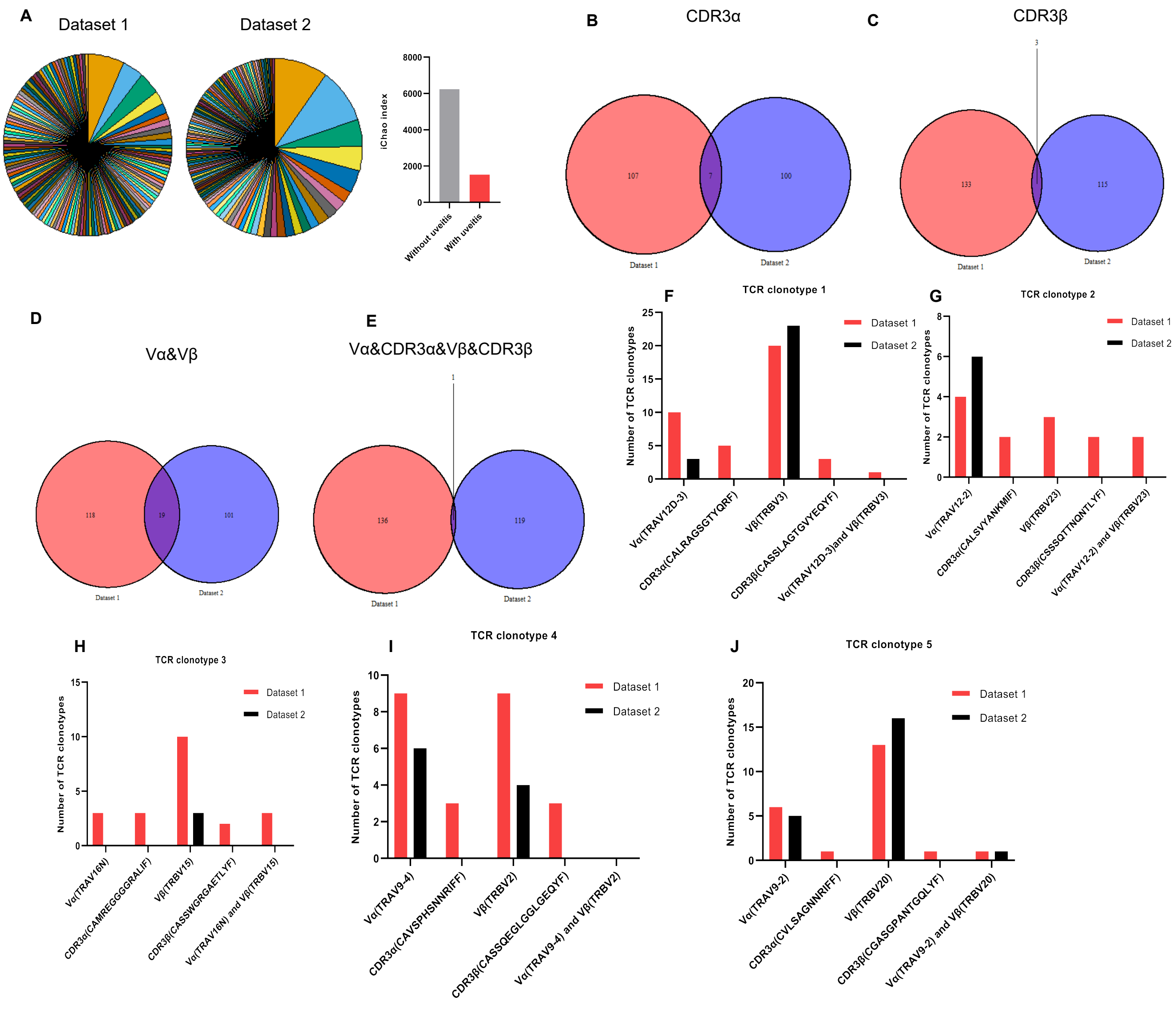

A
Dataset 1
Dataset 2
iChao index
8000
6000
4000
2000
0
Without uveitis
With uveitis
B
CDR3α
107
7
100
Dataset 1
Dataset 2
C
CDR3β
3
133
115
Dataset 1
Dataset 2
D
Vα&Vβ
118
19
101
Dataset 1
Dataset 2
E
Vα&CDR3α&Vβ&CDR3β
1
136
119
Dataset 1
Dataset 2
F
TCR clonotype 1
Number of TCR clonotypes
Dataset 1
Dataset 2
Vα(TRAV12D-3)
CDR3α(CALRAGSGTYQRF)
Vβ(TRBV3)
CDR3β(CASSLAGTGVYEQYF)
Vα(TRAV12D-3)and Vβ(TRBV3)
G
TCR clonotype 2
Number of TCR clonotypes
Dataset 1
Dataset 2
Vα(TRAV12-2)
CDR3α(CALSVYANKMIF)
Vβ(TRBV23)
CDR3β(CSSSQTTNQNTLYF)
Vα(TRAV12-2) and Vβ(TRBV23)
H
TCR clonotype 3
Number of TCR clonotypes
Dataset 1
Dataset 2
Vα(TRAV16N)
CDR3α(CAMREGGGGRALIF)
Vβ(TRBV15)
CDR3β(CASSWGRGAETLYF)
Vα(TRAV16N) and Vβ(TRBV15)
I
TCR clonotype 4
Number of TCR clonotypes
Dataset 1
Dataset 2
Vα(TRAV9-4)
CDR3α(CAVSPHSNNRIFF)
Vβ(TRBV2)
CDR3β(CASSQEGLGGLGEQYF)
Vα(TRAV9-4) and Vβ(TRBV2)
J
TCR clonotype 5
Number of TCR clonotypes
Dataset 1
Dataset 2
Vα(TRAV9-2)
CDR3α(CVLSAGNNRIFF)
Vβ(TRBV20)
CDR3β(CGASGPANTGQLYF)
Vα(TRAV9-2) and Vβ(TRBV20)

Fig. S1. TCR clonotypes in P2^{+}CD4^{+} T cells in LN of Aire$^{GW/+}$Lyn$^{-/-}$ mice. (A) Relative abundance of TCR clonotypes in dataset 1 (left: 170 paired TCRα and TCRβ sequences) and in dataset 2 (middle: 246 paired sequences) of Aire$^{GW/+}$Lyn$^{-/-}$ mice with uveitis. There were 137 clonotypes within dataset 1 and 120 clonotypes within dataset 2. Right: iChao index for TCR clonotypes in the P2^{+}CD4^{+}T cells in LN of Aire$^{GW/+}$Lyn$^{-/-}$ mice without uveitis and with uveitis (combination of two datasets).(**B** to **E**) Number of clonotypes that overlap between the two datasets for the listed parameter (B to D) or exactly in Vα and Vβ amino acid sequences (E). (**F** to **J**) The number of clonotypes from dataset 1 (red) and dataset 2 (black) matching the identified TCR clonotype with regard to Vα, CDR3α, Vβ, CDR3β, or paired Vα / Vβ.

Fig. S2

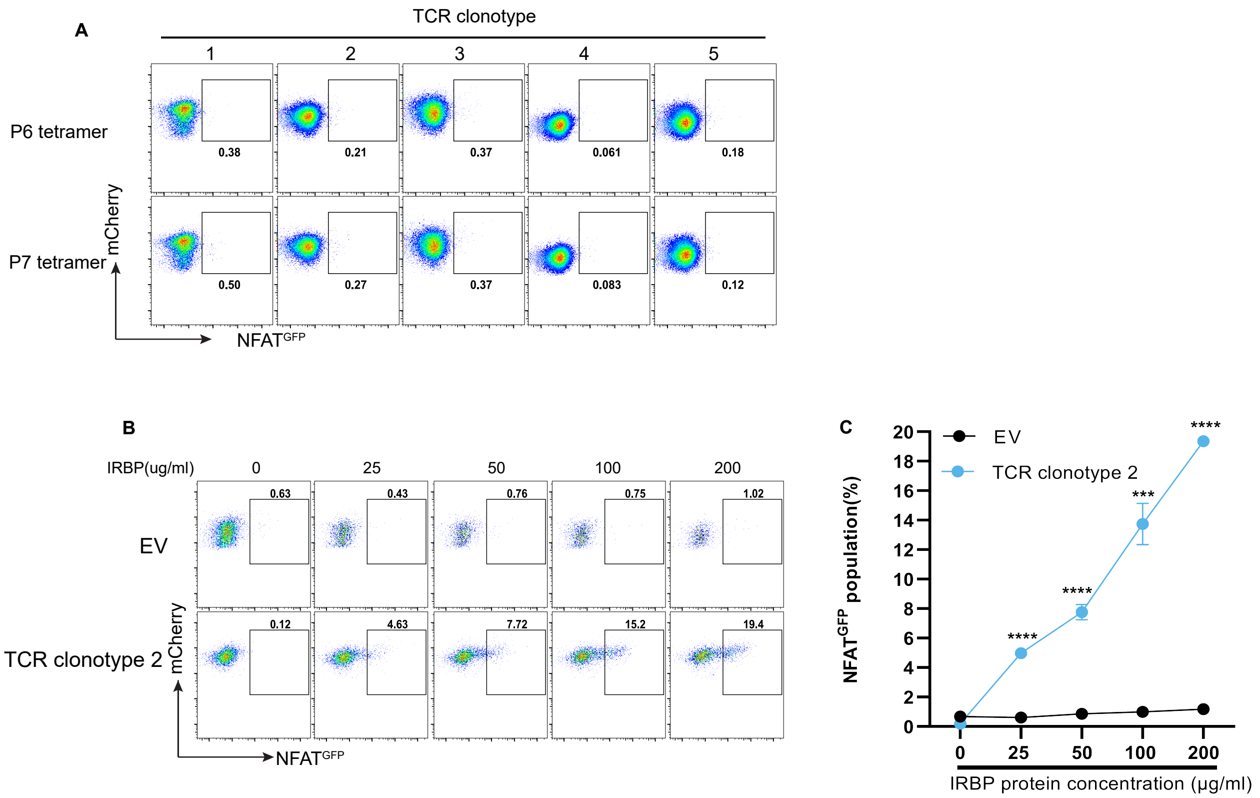

A
TCR clonotype
1
2
3
4
5
P6 tetramer
0.38
0.21
0.37
0.061
0.18
P7 tetramer
0.50
0.27
0.37
0.083
0.12
mCherry
NFAT^GFP
B
IRBP(ug/ml)
0
25
50
100
200
EV
0.63
0.43
0.76
0.75
1.02
TCR clonotype 2
0.12
4.63
7.72
15.2
19.4
mCherry
NFAT^GFP
C
EV
TCR clonotype 2
NFAT^GFP population(%)
20
18
16
14
12
10
8
6
4
2
0
****
****
****
***
****
0
25
50
100
200
IRBP protein concentration (μg/ml)

Fig. S2. Hybridoma cells expressing TCR clonotype 2 fail to respond to other IRBP peptide-containing tetramers, but do respond to APC incubated with intact IRBP. (**A**) Transfected hybridomas cells expressing TCR clonotypes 1,2,3,4, or 5 and mCherry were stimulated with P6 tetramer (IRBP amino acids 654–664) or P7 tetramer (IRBP amino acids 771–782) for 24 h and analyzed for NFAT-GFP expression by flow cytometry. (**B**) Transfected hybridomas cells expressing empty vector (EV) or TCR clonotype 2 were stimulated for 24h with different doses of IRBP protein in the presence of DCs from Lyn$^{-/-}$ mice and analyzed for NFAT-GFP induction by flow cytometry. (**C**) Frequency of GFP expression in (B**)**. ***$P < 0.001$; ****$p < 0.0001$. Two-tailed t-test; error bars are mean ± SD. Data are representative of three independent experiments.

Fig. S3

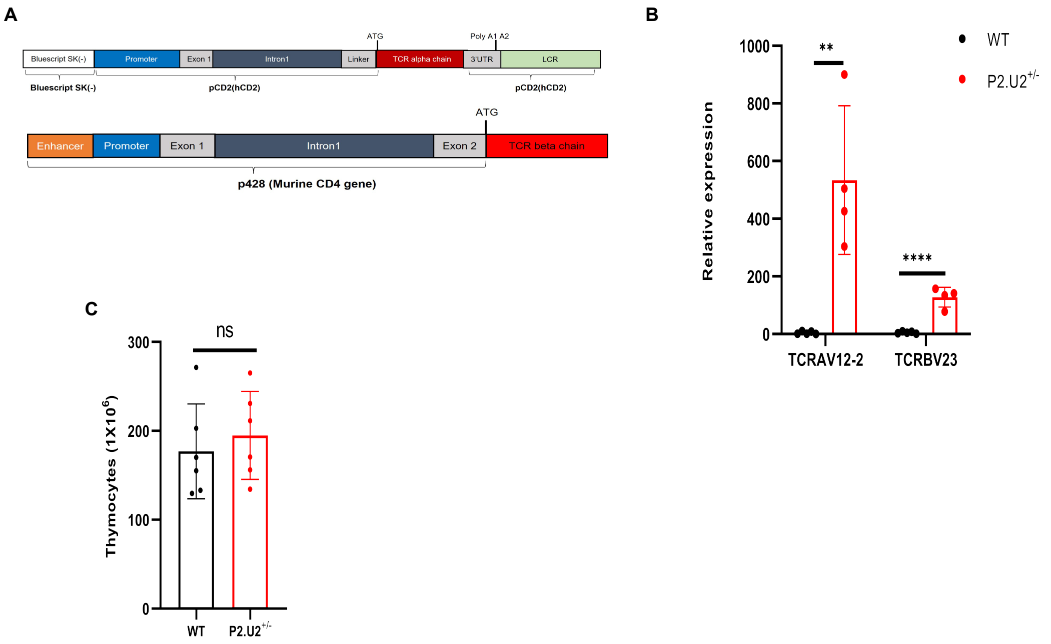
A
ATG
Poly A1 A2
Bluescript SK(-)
Promoter
Exon 1
Intron1
Linker
TCR alpha chain
3'UTR
LCR
Bluescript SK(-)
pCD2(hCD2)
pCD2(hCD2)
ATG
Enhancer
Promoter
Exon 1
Intron1
Exon 2
TCR beta chain
p428 (Murine CD4 gene)
B
WT
P2.U2+/-
Relative expression
1000
800
600
400
200
0
**
****
TCRAV12-2
TCRBV23
C
ns
Thymocytes (1X10^6)
300
200
100
0
WT
P2.U2+/-

Fig. S3. Elevated expression of transgenic TCR clonotype 2 Vα and Vβ mRNA in P2.U2$^{+/-}$ mice. (**A**) Schematic of the pCD2 and p428 vectors used to make the P2.U2 transgenic mice. Upper: Schematic of the pCD2 vector. The pCD2 vector was used for expression of the TCRα chain from elements of the human CD2 (hCD2) minigene. pCD2 vector included from the hCD2 locus the promoter, exon I with ATG start codon mutated, intron 1, polyA1 and A2 addition sites, 3'UTR untranslated region (3'UTR) and locus control region (LCR), in addition to some vector sequences of the Bluescript SK(-) plasmid. Lower: Schematic of the p428 vector. The p428 vector was used for expression of the TCRβ chain from elements of the mouse CD4 (mCD4) locus. The p428 vector included the enhancer, promoter, untranslated exon I, intron I and part of exon II of mCD4. (**B**) Relative expression of TCRAV12-2 and TCRBV23 sequences within mRNA of thymocytes in WT(n=5) and P2.U2$^{+/-}$(n=4) mice, as determined by real-time quantitative PCR. (**C**) Quantification of the number of thymocytes in WT(n=6) and P2.U2$^{+/-}$ (n=6) mice at 5-7 weeks of age. Data were pooled from at least three independent experiments. ns: Not Significant. ** $P<0.01$; ****$P<0.0001$. Two-tailed t-test; error bars are mean ± SD.

Fig. S4

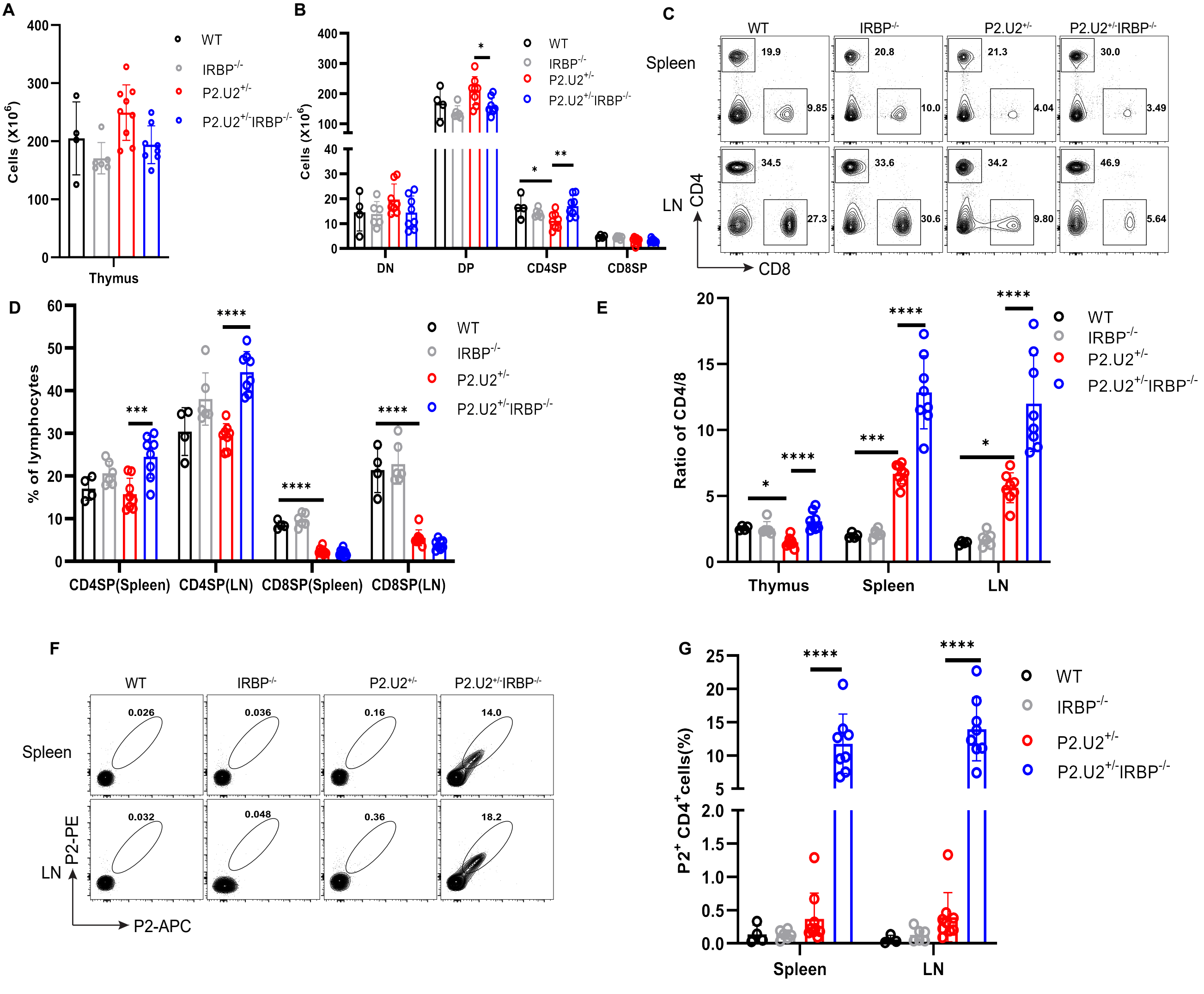
A
Cells (X10^6)
Thymus
WT
IRBP-/-
P2.U2+/-
P2.U2+/-IRBP-/-
B
Cells (X10^6)
DN
DP
CD4SP
CD8SP
C
WT
IRBP-/-
P2.U2+/-
P2.U2+/-IRBP-/-
Spleen
LN
CD4
CD8
19.9
9.85
20.8
10.0
21.3
4.04
30.0
3.49
34.5
27.3
33.6
30.6
34.2
9.80
46.9
5.64
D
% of lymphocytes
CD4SP(Spleen)
CD4SP(LN)
CD8SP(Spleen)
CD8SP(LN)
E
Ratio of CD4/8
Thymus
Spleen
LN
F
Spleen
LN
P2-PE
P2-APC
0.026
0.036
0.16
14.0
0.032
0.048
0.36
18.2
G
P2+ CD4+cells(%)
Spleen
LN

Fig. S4. Effect of IRBP expression on numbers of P2.U2 TCR transgenic T cells in thymus, spleen and LN. Additional data related to the data shown in Fig. 4. (**A**) Total numbers of thymocytes in WT (n=4), $IRBP^{-/-}$(n=6), $P2.U2^{+/-}$ (n=9) and $P2.U2^{+/-}IRBP^{-/-}$ (n=8) mice at 5-7 weeks of age. (**B**) Numbers of DN, DP, CD4SP and CD8SP thymocytes in WT(n=4), $IRBP^{-/-}$(n=6), $P2.U2^{+/-}$ (n=8) and $P2.U2^{+/-}IRBP^{-/-}$ (n=8) mice at 5-7 weeks of age. (**C** and **D**) Representative flow cytometric analysis (C) and frequencies (D) of CD4SP and CD8SP T cells in spleen and LN of WT(n=4), $IRBP^{-/-}$(n=6), $P2.U2^{+/-}$ (n=8) and $P2.U2^{+/-}IRBP^{-/-}$ (n=8) mice at 5-7 weeks of age. (**E**) Summary for the ratio of CD4SP to CD8SP T cells ($CD4^{+}CD8^{-}/CD4^{-}CD8^{+}$) in thymus, spleen and LN. (**F** and **G**) Representative flow cytometric analysis (F) and frequencies (G) of $P2^{+}$ cells in spleen and LN of WT (LN(n=3), spleen(n=4)), $IRBP^{-/-}$(n=6), $P2.U2^{+/-}$ (n=9) and $P2.U2^{+/-}IRBP^{-/-}$ (n=8) mice at 5-7 weeks. Cells were gated on the $TCR\beta^{+}CD4^{+}CD8^{-}DUMP^{-}$ cells. Data in the panels were pooled from at least three independent experiments. In (A, D, E and G), one-way ANOVA with Tukey's multiple comparisons tests were used. In (B), two-tailed t-tests were used. *p < 0.05; **p < 0.01; ***p < 0.001; ****p < 0.0001. Error bars are mean ± SD.

Fig. S5

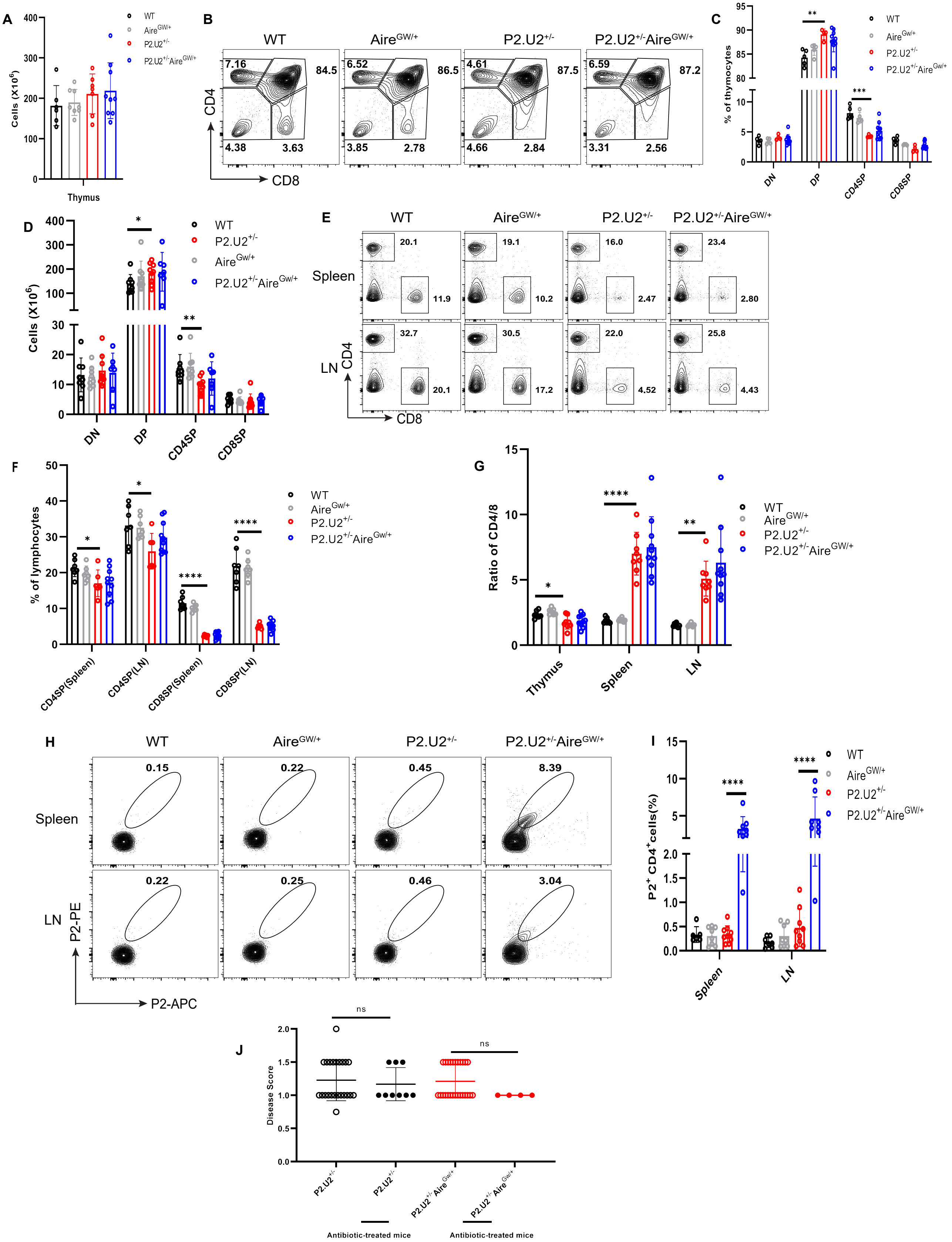
A
Cells (X10^6)
Thymus
WT
Aire^GW/+
P2.U2^+/-
P2.U2^+/-Aire^GW/+
B
WT
Aire^GW/+
P2.U2^+/-
P2.U2^+/-Aire^GW/+
CD4
CD8
7.16
84.5
4.38
3.63
6.52
86.5
3.85
2.78
4.61
87.5
4.66
2.84
6.59
87.2
3.31
2.56
C
% of thymocytes
DN
DP
CD4SP
CD8SP
D
Cells (X10^6)
DN
DP
CD4SP
CD8SP
E
Spleen
LN
20.1
11.9
19.1
10.2
16.0
2.47
23.4
2.80
32.7
20.1
30.5
17.2
22.0
4.52
25.8
4.43
F
% of lymphocytes
CD4SP(Spleen)
CD4SP(LN)
CD8SP(Spleen)
CD8SP(LN)
G
Ratio of CD4/8
Thymus
Spleen
LN
H
P2-PE
P2-APC
0.15
0.22
0.45
8.39
0.22
0.25
0.46
3.04
I
P2^+ CD4^+cells(%)
Spleen
LN
J
Disease Score
ns
P2.U2^+/-
P2.U2^+/- Aire^GW/+
Antibiotic-treated mice

Fig. S5. Development of P2.U2 transgenic T cells in Aire$^{GW/+}$mice. (**A**) Quantification of thymocytes in WT (n=6), Aire$^{GW/+}$ (n=8), P2.U2$^{+/-}$ (n=7) and P2.U2$^{+/-}$Aire$^{GW/+}$ (n=9) mice at 5-7 weeks of age. For WT group of this figure, the data of 3 out of 6 mice is the same with the data of 3 out of 6 mice in WT group of Fig. S3C. For P2.U2$^{+/-}$ group of this figure, the data of 4 out of 7 mice is the same with the data of 4 out of 6 mice in P2.U2$^{+/-}$ group of Fig. S3C. (**B** and **C**) Representative flow cytometric analysis (B) and frequencies (C) of DN (CD4^{-}CD8^{-}), DP (CD4^{+}CD8^{+}), CD4SP (CD4^{+}CD8^{-}) and CD8SP(CD4^{-}CD8^{+}) cells in thymus of WT (n=5), Aire$^{GW/+}$ (n=5), P2.U2$^{+/-}$ (n=4) and P2.U2$^{+/-}$Aire$^{GW/+}$ (n=10) mice at 5-7 weeks of age. (**D**) Quantification of DN, DP, CD4SP and CD8SP thymocytes in WT (n=8), Aire$^{GW/+}$ (n=8), P2.U2$^{+/-}$ (n=9) and P2.U2$^{+/-}$Aire$^{GW/+}$ (n=7) mice at 5-7 weeks of age. For WT group of this figure, the data of 6 out of 8 mice are the same with the data of WT group of Fig. 3B (right panel). For P2.U2$^{+/-}$ group of this figure, the data of 6 out of 9 mice are the same with the data of P2.U2$^{+/-}$ group of Fig. 3B (right panel). (**E** and **F**) Representative flow cytometric analysis (E) and frequencies (F) of CD4SP and CD8SP T cells in spleen and LN of WT (n=7), Aire$^{GW/+}$ (n=7), P2.U2$^{+/-}$ (n=6) and P2.U2$^{+/-}$Aire$^{GW/+}$ (n=10) mice at 5-7 weeks of age. For WT group of Fig. S5F, the data of 2 out of 7 mice are the same with the data of 2 out of 6 mice for WT group of Fig. 3C. For P2.U2$^{+/-}$ group of Fig. S5F, the data of 2 out of 6 mice are the same with the data of 2 out of 6 mice for P2.U2$^{+/-}$ group of Fig. 3C. (**G**) Summary for the ratio of CD4^{+}CD8^{-}/CD4^{-}CD8^{+} T cells in thymus, LN and spleens of WT (n=7), Aire$^{GW/+}$ (n=7), P2.U2$^{+/-}$ (n=8) and P2.U2$^{+/-}$Aire$^{GW/+}$ (n=10) mice at 5-7 weeks of age. For WT group of Fig. S5G, the data of 2 out of 7 mice are the same with the data of 2 out of 6 mice for WT group of Fig. 3D. For P2.U2$^{+/-}$ group of Fig. S5G, the data of 4 out of 8 mice are the same with the data of 4 out of 6 mice for P2.U2$^{+/-}$ group of Fig. 3D. (**H** and **I**) Representative flow cytometric analysis (H) and frequencies (I) of P2^{+}CD4^{+} T cells in spleen and LN of WT (spleen:n=6; LN:n=7), Aire$^{GW/+}$ (n=8), P2.U2$^{+/-}$ (n=9) and P2.U2$^{+/-}$Aire$^{GW/+}$ (n=8) mice at 5-7 weeks. Cells were gated on the TCRβ^{+}CD4^{+}CD8^{-}DUMP^{-} cells. Data were pooled from at least three independent experiments. (**J**) Individual disease score of funduscopic images with uveitis for P2.U2$^{+/-}$ (without antibiotic treatment: n=23; with antibiotic treatment: n=9) and P2.U2$^{+/-}$Aire$^{GW/+}$ (without antibiotic treatment: n=26; with antibiotic treatment: n=4)mice without and with antibiotic treatment in Fig.5I and Fig.5K. Disease severity was scored on a scale of 0-3 based on number, type, size, and pattern of lesions: 0 = normal, 1 = mild, 1.5=mild to moderate between 1 and 2, 2 = moderate, 3 = severe. In (A, C, G and I), one-way ANOVA with Tukey's multiple comparisons tests were used. In (D, F and J), two-tailed t-tests were used. ns: Not Significant. *p < 0.05; **p < 0.01; ***p < 0.001; ****p < 0.0001.

Fig. S6

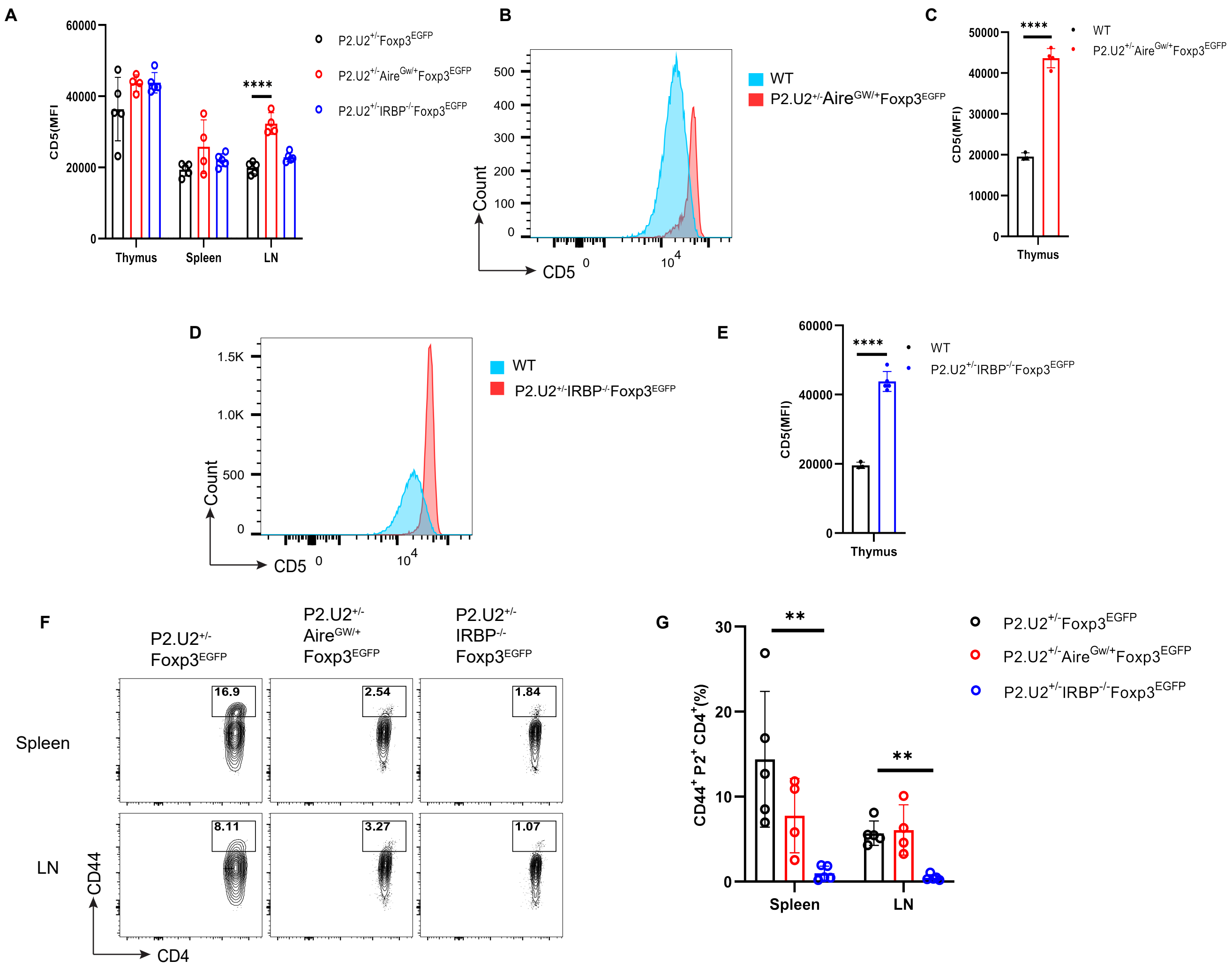
A
60000
40000
20000
0
CD5(MFI
****
Thymus
Spleen
LN
P2.U2+/-Foxp3EGFP
P2.U2+/-AireGw/+Foxp3EGFP
P2.U2+/-IRBP-/-Foxp3EGFP
B
500
400
300
200
100
0
Count
CD5
0
10^4
WT
P2.U2+/-AireGW/+Foxp3EGFP
C
50000
40000
30000
20000
10000
0
CD5(MFI)
****
Thymus
WT
P2.U2+/-AireGw/+Foxp3EGFP
D
1.5K
1.0K
500
0
Count
CD5
0
10^4
WT
P2.U2+/-IRBP-/-Foxp3EGFP
E
60000
40000
20000
0
CD5(MFI)
****
Thymus
WT
P2.U2+/-IRBP-/-Foxp3EGFP
F
P2.U2+/- Foxp3EGFP
P2.U2+/- AireGW/+ Foxp3EGFP
P2.U2+/- IRBP-/- Foxp3EGFP
Spleen
LN
16.9
2.54
1.84
8.11
3.27
1.07
CD44
CD4
G
30
20
10
0
CD44+ P2+ CD4+(%)
**
**
Spleen
LN
P2.U2+/-Foxp3EGFP
P2.U2+/-AireGw/+Foxp3EGFP
P2.U2+/-IRBP-/-Foxp3EGFP

Fig. S6. Expression of CD5 and CD44 by transgenic T cells in thymus, spleen and LN. (**A**) MFI of CD5 expression on P2^{+}CD4^{+}T cells in the thymus, spleen and LN of P2.U2$^{+/-}$Foxp3^{EGFP} (n=5), P2.U2$^{+/-}$Aire$^{GW/+}$Foxp3^{EGFP} (n=4) and P2.U2$^{+/-}$IRBP$^{-/-}$Foxp3^{EGFP} (n=5) mice at 6-10 weeks of age. Cells were gated on TCRβ^{+}CD4^{+}CD8^{-}DUMP^{-} P2^{+}cells. (**B**) Overlaid histograms show the expression of CD5 in P2^{-}CD4^{+}T cell in thymus of WT and P2^{+}CD4^{+}T cells in thymus of P2.U2$^{+/-}$Aire$^{GW/+}$Foxp3^{EGFP} mice at 6-10 weeks of age. Cells were gated on TCRβ^{+}CD4^{+}CD8^{-}DUMP^{-} P2^{-}cells in WT mice. Cells were gated on TCRβ^{+}CD4^{+}CD8^{-}DUMP^{-} P2^{+}cells in P2.U2$^{+/-}$Aire$^{GW/+}$Foxp3^{EGFP} mice. (**C**) MFI of CD5 in P2^{-}CD4^{+}T cell in thymus of WT(n=3) and P2^{+}CD4^{+}T cells in thymus of P2.U2$^{+/-}$Aire$^{GW/+}$Foxp3^{EGFP} (n=4) mice in panel B. (**D**) Overlaid histograms show the expression of CD5 in P2^{-}CD4^{+}T cell in thymus of WT and P2^{+}CD4^{+}T cells in thymus of P2.U2$^{+/-}$IRBP$^{-/-}$Foxp3^{EGFP} mice at 6-10 weeks of age. Cells were gated on TCRβ^{+}CD4^{+}CD8^{-}DUMP^{-} P2^{-}cells in WT mice. Cells were gated on TCRβ^{+}CD4^{+}CD8^{-}DUMP^{-} P2^{+}cells in P2.U2$^{+/-}$IRBP$^{-/-}$Foxp3^{EGFP} mice. (**E**) MFI of CD5 in P2^{-}CD4^{+}T cell in thymus of WT(n=3) and P2^{+}CD4^{+}T cells in thymus of P2.U2$^{+/-}$IRBP$^{-/-}$Foxp3^{EGFP} (n=5) mice in panel D. (**F-G**) Representative flow cytometric analysis (F) and frequencies (G) of CD44^{+}P2^{+}CD4^{+}T cells in spleen and LN of P2.U2$^{+/-}$Foxp3^{EGFP} (n=5), P2.U2$^{+/-}$Aire$^{GW/+}$Foxp3^{EGFP} (n=4) and P2.U2$^{+/-}$IRBP$^{-/-}$Foxp3^{EGFP} (n=5) mice at 6-10 weeks of age. Cells were gated on TCRβ^{+}CD4^{+}CD8^{-}DUMP^{-} P2^{+}cells. $^{**}p < 0.01$; $^{****}p < 0.0001$. In (A and G), one-way ANOVA with Tukey's multiple comparisons tests were used. In (C and E), two-tailed t-test; Error bars are mean ± SD.

Fig. S7

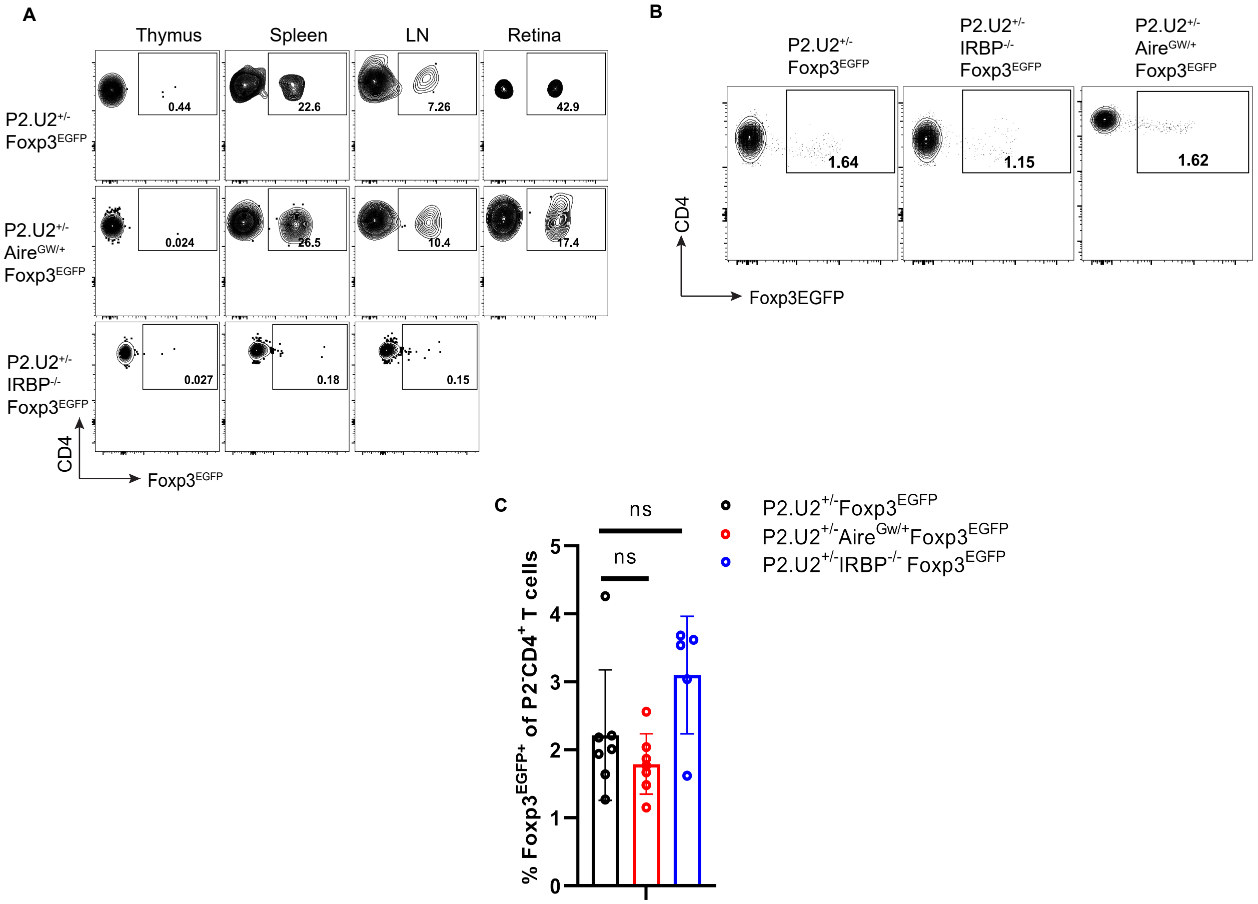
A
Thymus
Spleen
LN
Retina
P2.U2+/- Foxp3EGFP
0.44
22.6
7.26
42.9
P2.U2+/- AireGW/+ Foxp3EGFP
0.024
26.5
10.4
17.4
P2.U2+/- IRBP-/- Foxp3EGFP
0.027
0.18
0.15
CD4
Foxp3EGFP
B
P2.U2+/- Foxp3EGFP
P2.U2+/- IRBP-/- Foxp3EGFP
P2.U2+/- AireGW/+ Foxp3EGFP
1.64
1.15
1.62
CD4
Foxp3EGFP
C
ns
ns
% Foxp3EGFP+ of P2-CD4+ T cells
0
1
2
3
4
5
P2.U2+/-Foxp3EGFP
P2.U2+/-AireGW/+Foxp3EGFP
P2.U2+/-IRBP-/- Foxp3EGFP

Fig. S7. P2-binding and Non P2-binding Tregs in P2.U2$^{+/-}$IRBP$^{-/-}$Foxp3^{EGFP} and P2.U2$^{+/-}$Aire$^{GW/+}$Foxp3^{EGFP} mice. (**A**) Representative flow cytometric analysis of Foxp3^{EGFP+} of P2^{+} CD4^{+}T cells in the thymus, spleen, LN and retina of P2.U2$^{+/-}$Foxp3^{EGFP}, P2.U2$^{+/-}$Aire$^{GW/+}$Foxp3^{EGFP} and P2.U2$^{+/-}$IRBP$^{-/-}$Foxp3^{EGFP} mice at 6-7 weeks of age for Fig. 6. Cells shown were gated on TCRβ^{+}CD4^{+}CD8^{-}DUMP^{-}P2^{+} cells. (**B** and **C**) Representative flow cytometric analysis (**B**) and frequencies (**C**) of Foxp3^{EGFP+} in non-P2 tetramer binding CD4^{+}T cells in the thymus of P2.U2$^{+/-}$Foxp3^{EGFP} (n=7), P2.U2$^{+/-}$Aire$^{GW/+}$Foxp3^{EGFP} (n=7) and P2.U2$^{+/-}$IRBP$^{-/-}$Foxp3^{EGFP} (n=5) mice at 5-7 weeks of age. Cells were gated on TCRβ^{+}CD4^{+}CD8^{-}DUMP^{-} P2^{-}cells. In (C), one-way ANOVA with Tukey's multiple comparisons tests were used; ns: Not Significant. error bars are mean ± SD.

Fig. S8

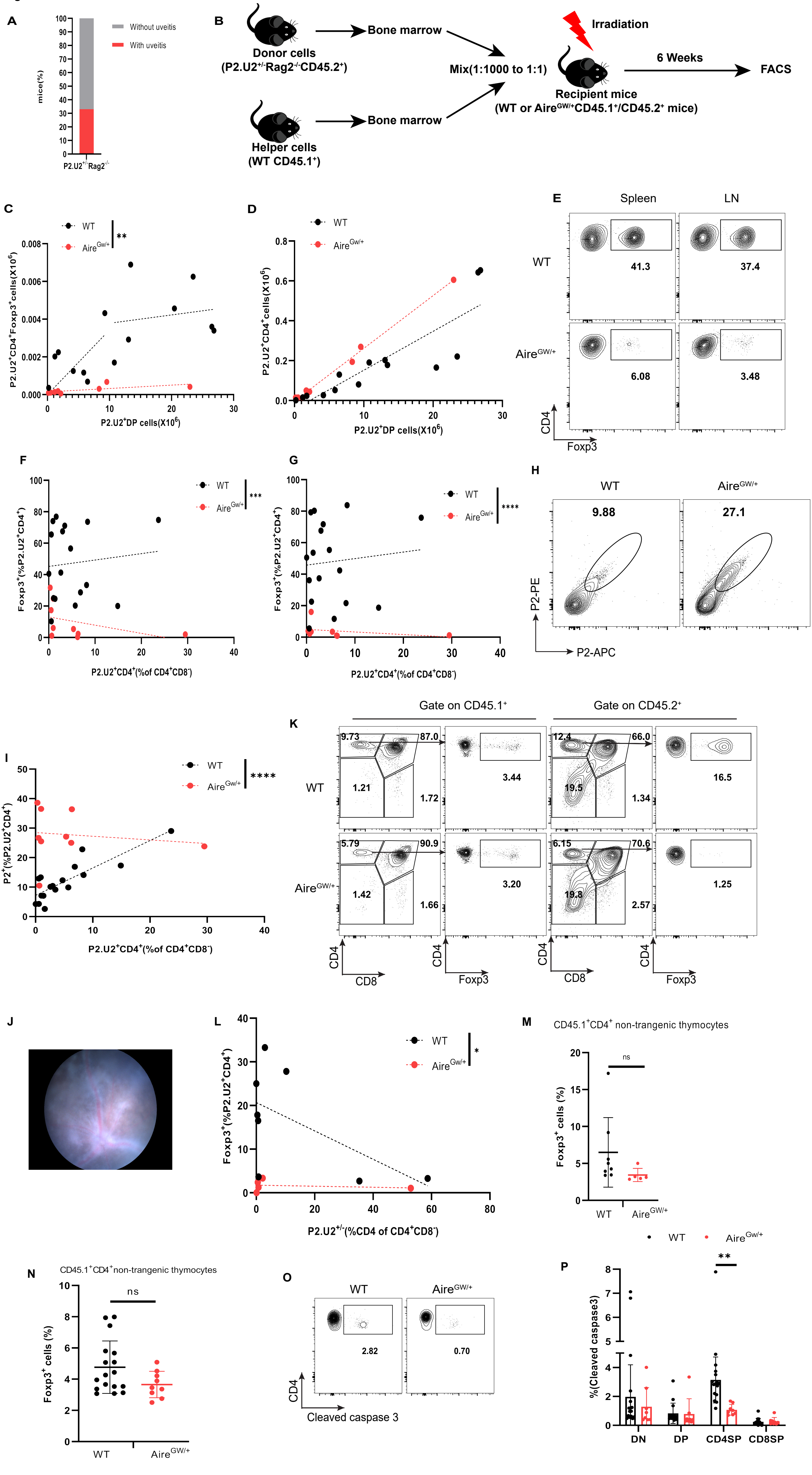
A
Without uveitis
With uveitis
mice(%)
P2.U2+/-Rag2-/-
B
Donor cells
(P2.U2+/-Rag2-/-CD45.2+)
Bone marrow
Helper cells
(WT CD45.1+)
Bone marrow
Mix(1:1000 to 1:1)
Irradiation
Recipient mice
(WT or AireGW/+CD45.1+/CD45.2+ mice)
6 Weeks
FACS
C
WT
AireGw/+
**
P2.U2+CD4+Foxp3+cells(X10^6)
P2.U2+DP cells(X10^6)
D
WT
AireGw/+
P2.U2+CD4+cells(X10^6)
P2.U2+DP cells(X10^6)
E
Spleen
LN
WT
41.3
37.4
AireGW/+
6.08
3.48
CD4
Foxp3
F
WT
AireGw/+
***
Foxp3+(%P2.U2+CD4+)
P2.U2+CD4+(%of CD4+CD8-)
G
WT
AireGw/+
****
Foxp3+(%P2.U2+CD4+)
P2.U2+CD4+(%of CD4+CD8-)
H
WT
AireGW/+
9.88
27.1
P2-PE
P2-APC
I
WT
AireGw/+
****
P2+(%P2.U2+CD4+)
P2.U2+CD4+(%of CD4+CD8-)
K
Gate on CD45.1+
Gate on CD45.2+
WT
9.73
87.0
1.21
1.72
3.44
12.4
66.0
19.5
1.34
16.5
AireGW/+
5.79
90.9
1.42
1.66
3.20
6.15
70.6
19.8
2.57
1.25
CD4
CD8
CD4
Foxp3
CD4
CD8
CD4
Foxp3
J
L
WT
AireGw/+
*
Foxp3+(%P2.U2+CD4+)
P2.U2+/-(%CD4 of CD4+CD8-)
M
CD45.1+CD4+ non-trangenic thymocytes
ns
Foxp3+ cells (%)
WT
AireGW/+
N
CD45.1+CD4+non-trangenic thymocytes
ns
Foxp3+ cells (%)
WT
AireGW/+
O
WT
AireGW/+
2.82
0.70
CD4
Cleaved caspase 3
P
WT
AireGw/+
**
%(Cleaved caspase3)
DN
DP
CD4SP
CD8SP

Fig. S8. Aire-dependent thymic development of IRBP P2 antigen-specific Tregs. Analysis of Treg numbers of P2.U2$^{+/-}$ TCR transgenic T cells in thymus, spleen and LN and additional analysis related to the mixed bone marrow chimeric mice analyzed in Fig. 7. (**A**) Frequencies of uveitis or lack of uveitis in P2.U2$^{+/-}$Rag2$^{-/-}$ (n=30) mice at 1-5 months of age. (**B**) Experimental schematic for Fig. 7. Mixed bone marrow chimeric mice were made in which bone marrow cells of P2.U2$^{+/-}$Rag2$^{-/-}$CD45.2^{+} male donor mice were engrafted along with various fractions of bone marrow cells(helper cells) from male WT CD45.1^{+} mice. Recipient mice were irradiated WT or Aire$^{GW/+}$CD45.1^{+}/CD45.2^{+} mice. Six weeks post-engraftment, the fate of P2.U2^{+} cells was analyzed by using FACS. (**C**) Comparison of the number of P2.U2$^{+/-}$Rag2$^{-/-}$CD45.2^{+}CD4^{+} Foxp3-expressing Treg in the thymus of individual mixed bone marrow chimeric mice to the number of P2.U2$^{+/-}$Rag2$^{-/-}$CD45.2^{+}DP thymocytes in recipient mice of the indicated genotype from the experiments shown in Fig. 7A-C. Dashed lines indicate nonlinear regression (curve fit) line (WT(left, n=7): R= 0.074; WT(right, n=7): R= 0.026; Aire$^{GW/+}$(n=8): R= 0.450). (**D**) Comparison of the number of P2.U2$^{+/-}$Rag2$^{-/-}$CD45.2^{+}CD4^{+} CD8^{-} cells in the thymus of individual mixed bone marrow chimeric mice to the number of P2.U2$^{+/-}$Rag2$^{-/-}$CD45.2^{+}DP thymocytes in recipient mice of the indicated genotype from the experiments shown in Fig. 7A-C. Dashed lines indicate simple linear regression lines (WT(n=14): R= 0.749; Aire$^{GW/+}$(n=8): R= 0.995). (**E**) Representative flow cytometric analysis of CD45.2^{+}P2.U2^{+}Rag2$^{-/-}$T cells in spleen and LN from the indicated Aire genotype recipient mice. Cells were gated on the CD45.2^{+}CD45.1^{-}CD4^{+}CD8^{-} cells. (**F** and **G**) Summary plots of the frequencies of P2.U2^{+} CD4^{+} T cells that expressed Foxp3 (y-axis) in spleen (F) or LN (G), relative to frequencies of P2.U2$^{+/-}$Rag2$^{-/-}$CD45.2^{+}CD4^{+} thymocytes in all donor CD4^{+}CD8^{-} cells isolated from recipient mice of the indicated genotype (represented on the x-axis). Dashed lines indicate simple linear regression line (for F: WT(n=17): R= 0.011; Aire$^{GW/+}$(n=9): R= 0.176, for G: WT(n=17):R=0.010; Aire$^{GW/+}$(n=9): R=0.106). (**H**) Representative flow cytometric analysis of CD45.2^{+}P2.U2^{+} Rag2$^{-/-}$P2^{+}CD4^{+}T cells in thymus from the indicated Aire genotype recipient mice. Cells were gated on the CD45.2^{+}CD45.1^{-}TCRβ^{+}CD4^{+}CD8^{-}DUMP^{-}thymocytes. (**I**) Summary plots of the frequencies of CD45.2^{+}P2.U2^{+}Rag2$^{-/-}$ P2^{+}CD4^{+} T cells relative to frequencies of P2.U2$^{+/-}$Rag2$^{-/-}$CD45.2^{+}CD4^{+} thymocytes in all donor CD4^{+}CD8^{-} cells isolated from recipient mice of the indicated genotype (represented on the x-axis). Dashed lines indicate simple linear regression line (WT(n=17):R=0.709; Aire$^{GW/+}$(n=9): R=0.018). **(J**) Representative funduscopic image of Aire$^{GW/+}$CD45.1^{+}/CD45.2^{+}recipient mice with uveitis. (**K** to **M**) Bone marrow cells from P2.U2$^{+/-}$CD45.2^{+} female donor mice were engrafted, along with polyclonal "helper"cells from females (CD45.1^{+}), into irradiated WT or Aire$^{GW/+}$CD45.1^{+}/CD45.2^{+}recipient mice. Six weeks post-engraftment, the fate of P2.U2^{+} cells was analyzed. (**K**) Representative flow cytometric analysis of CD45.1^{+}polyclonal T cells (left) and CD45.2^{+} P2.U2^{+} T cells (right) from recipient mice of the indicated Aire genotype. The frequencies of CD4^{+} Foxp3^{+} cells are shown. For the thymus, the left column (CD4 versus CD8) depicts undepleted samples; the right column (Foxp3 versus CD4) depicts CD8 depleted samples. (**L**) Summary plots of the efficiency of P2.U2^{+} Treg development, in which the frequencies of P2.U2$^{+/-}$CD45.2^{+} CD4^{+}T cells that express Foxp3 is plotted versus the frequency of P2.U2$^{+/-}$CD45.2^{+}CD4^{+} thymocytes (as a percentage of all CD4^{+}CD8^{-}cells) for cells isolated from

recipient mice of the indicated genotype. Dashed lines indicate simple linear regression line (WT(n=8):R=0.346; $Aire^{GW/+}$(n=5)-:R=0.045). (**M**) Frequencies of $CD45.1^{+}$ polyclonal CD4SP thymocytes that express Foxp3 in chimeric mice of WT(n=8) or $Aire^{GW/+}$ (n=5) recipient in panel K, L. ns: Not Significant. (**N**) Frequencies of $CD45.1^{+}$ polyclonal CD4SP thymocytes that express Foxp3 in chimeric mice of WT(n=17) or $Aire^{GW/+}$ (n=9) recipients in Fig. 7A. ns: Not Significant. (**O**) Representative flow cytometric analysis of cleaved caspase 3 in thymocytes of the indicated Aire genotype recipient mice in Fig. 7A, gated on the $CD45.2^{+}CD45.1^{-}CD4^{+}CD8^{-}$ cells. (**P**) Frequencies of cleaved caspase 3 in $CD45.2^{+}CD45.1^{-}$ DN, DP, CD4SP and CD8SP thymocytes (WT:n=16; $Aire^{GW/+}$:n=8) in Fig. 7A. *$p < 0.05$; **$p < 0.01$; ***$p < 0.001$; ****$p < 0.0001$. In (C, F, G, I, L, M, N and P), two-tailed t-test; Error bars are mean ± SD.
